# Computational modelling elucidates the mechanism of ciliary regulation in health and disease

**DOI:** 10.1186/1752-0509-5-143

**Published:** 2011-09-15

**Authors:** Nikolay V Kotov, Declan G Bates, Antonina N Gizatullina, Bulat Gilaziev, Rustem N Khairullin, Michael ZQ Chen, Ignat Drozdov, Yoshinori Umezawa, Christian Hundhausen, Alexey Aleksandrov, Xing-gang Yan, Sarah K Spurgeon, C Mark Smales, Najl V Valeyev

**Affiliations:** 1 Centre for Molecular Processing, School of Biosciences, University of Kent, Canterbury, Kent CT2 7NJ, UK; 2 Biophysics & Bionics Lab, Department of Physics, Kazan State University, Kazan 420008, Russia; 3 Centre for Systems, Dynamics and Control, College of Engineering, Mathematics and Physical Sciences, University of Exeter, Harrison Building, North Park Road, Exeter EX4 4QF, UK; 4 Inter-regional Diagnostic Centre, Karbisheva-12A, Kazan 420101, Russia; 5 Department of Mechanical Engineering, The University of Hong Kong, Pokfulam Road, Hong Kong; 6 School of Automation, Nanjing University of Science and Technology, 200 Xiao Ling Wei, Nanjing 210094, P. R. China; 7 Centre for Bioinformatics, Department of Computer Science, School of Physical Sciences & Engineering, King's College London, Strand, London WC2R 2LS, UK; 8 St. John's Institute of Dermatology, King's College London, 9th Floor Tower Wing, Guy's Hospital, Great Maze Pond, SE1 9RT London, UK; 9 Laboratoire de Biochimie, CNRS UMR7654, Department of Biology, Ecole Polytechnique, 91128 Palaiseau, France; 10 School of Engineering and Digital Arts, University of Kent, Canterbury, Kent CT2 7NT, UK

## Abstract

**Background:**

Ciliary dysfunction leads to a number of human pathologies, including primary ciliary dyskinesia, nephronophthisis, situs inversus pathology or infertility. The mechanism of cilia beating regulation is complex and despite extensive experimental characterization remains poorly understood. We develop a detailed systems model for calcium, membrane potential and cyclic nucleotide-dependent ciliary motility regulation.

**Results:**

The model describes the intimate relationship between calcium and potassium ionic concentrations inside and outside of cilia with membrane voltage and, for the first time, describes a novel type of ciliary excitability which plays the major role in ciliary movement regulation. Our model describes a mechanism that allows ciliary excitation to be robust over a wide physiological range of extracellular ionic concentrations. The model predicts the existence of several dynamic modes of ciliary regulation, such as the generation of intraciliary Ca^2+ ^spike with amplitude proportional to the degree of membrane depolarization, the ability to maintain stable oscillations, monostable multivibrator regimes, all of which are initiated by variability in ionic concentrations that translate into altered membrane voltage.

**Conclusions:**

Computational investigation of the model offers several new insights into the underlying molecular mechanisms of ciliary pathologies. According to our analysis, the reported dynamic regulatory modes can be a physiological reaction to alterations in the extracellular environment. However, modification of the dynamic modes, as a result of genetic mutations or environmental conditions, can cause a life threatening pathology.

## Background

Cilia are cellular protrusions which have been conserved in a wide range of organisms ranging from protozoa to the digestive, reproductive and respiratory systems of vertebrates [1]. Mobile or immotile cilia exist on every cell of the human body [2] and the insufficiently recognised importance of the cilium compartment in human physiology has been recently highlighted [1,3]. Cilia are present on most eukaryotic cell surfaces with the exception of the cells of higher plants and fungi [4]. Ciliary motility is important for moving fluids and particles over epithelial surfaces, and for the cell motility of vertebrate sperm and unicellular organisms. The cilium contains a microtubule-based axoneme that extends from the cell surface into the extracellular space. The axoneme consists of nine peripheral microtubule doublets arranged around a central core that may or may not contain two central microtubules (9+2 or 9+0 axoneme, respectively). Cilia can be broadly classified as 9+2 motile cilia or 9+0 immotile sensory cilia, although there are examples of 9+2 sensory cilia and 9+0 motile cilia. In mammals, motile 9+2 cilia normally concentrate in large numbers on the cell surface, beat in an orchestrated wavelike fashion, and are involved in fluid and cell movement. In contrast to motile cilia, primary cilia project as single immotile organelles from the cell surface. Primary cilia are found on nearly all cell types in mammals [5] and many are highly adapted to serve specialized sensory functions. The 9+2 cilia usually have dynein arms that link the microtubule doublets and are motile, while most 9+0 cilia lack dynein arms and are non-motile. In total, eight different types of cilia has been identified to date [6]. In this study, we investigate the mechanism of movement regulation for the motile type of cilia.

Although each individual cilium represents a tiny hair-like protrusion of only 0.25 *μm *in diameter and approximately 5-7 *μm *in length, cilia covering human airways can propel mucus with trapped particles of length up to 1 mm at a speed of 0.5 mm/second [7]. Such efficiency can be achieved due to the coordination between cilia and stimulus-dependent regulation of the rate of cilia beat. Dysfunction of ciliary regulation gives rise to pathologic phenotypes that range from being organ specific to broadly pleiotropic [3]. A link between ciliary function and human disease was discovered when individuals suffering from syndromes with symptoms including respiratory infections, anosmia, male infertility and situs inversus, were shown to have defects in ciliary structure and function [6].

Microscopic organisms that possess motile cilia which are used exclusively for either locomotion or to simply move liquid over their surface include *Paramecia*, *Karyorelictea*, *Tetrahymena*, *Vorticella *and others. The human mucociliary machinery operates in at least two different modes, corresponding to a low and high rate of beating. It has been shown that the high rate mode is mediated by second messengers [8], including purinergic, adrenergic and cholinergic receptors [9-19]. This mode enables a rapid response, which can last a significant period of time, to various stimuli by drastically increasing the ciliary beat frequency (CBF). At the same time, several ciliary movement modes have been reported in a ciliate *Paramecium caudatum *[20]. The remarkable conservation of ciliary mechanisms [21-25] creates grounds for the speculation that there can more than two ciliary beating modes in human tissues. It is, therefore, reasonable to suggest, that some human diseases, associated with aberrant ciliary motility, can arise due to modifications in the beating mode. Clearly, the development of therapeutic strategies against ciliary-associated pathologies will require advanced understanding of ciliary beating regulation mechanisms.

The periodic beating of cilia is governed by the internal apparatus of the organelle [26]. Its core part, the axoneme, contains nine microtubule pairs encircling the central pair. The transition at the junction of the cellular body and the ciliary axoneme is demarcated by Y-shaped fibres, which extend from the microtubule outer doublets to the ciliary membrane. The transition area, in combination with the internal structure of the basal body, is thought to function as a filter for the cilium, regulating the molecules that can pass into or out of the cilium. Ciliary motility is accomplished by dynein motor activity in a phosphorylation-dependent manner, which allows the microtubule doublets to slide relative to one another [12]. The dynein phosphorylation that controls ciliary activity is regulated by the interplay of calcium (Ca^2+^) and cyclic nucleotide pathways. The beating pattern of cilia consists of a fast effective stroke and a slower recovery stroke. During the effective stroke cilia are in an almost upright position, generating force for mucus movement. During the recovery stroke, the cilia are recovering from the power strike to the original position by moving in the vicinity of the cell surface.

Current theories which attempt to explain the workings of the Ca^2+^-dependent CBF regulation mechanism are incomplete and highly controversial. Elevation of intraciliary Ca^2+ ^is one of the major regulators of ciliary movement. Calcium influx regulates ciliary activity by increasing intraciliary Ca^2+ ^only, while the cytosolic bulk remains at a low level. Separate ciliary compartmentalisation for Ca^2+ ^allows prolonged activation of ciliary beating without damaging the cell through high Ca^2+ ^concentrations. It is well known that calcium fluxes via calcium channels lead to changes in organisms' swimming behaviour [27-29]. In mucus-transporting cilia, Ca^2+ ^mediates CBF increase [19,30-32]. It has also been shown that there are some differences in the Ca^2+^-dependent CBF regulation in single cell organisms and in humans [12]. Sustained CBF increase requires prolonged elevation of Ca^2+ ^levels which can be lethal to the cell [33,34]. It has been suggested that Ca^2+^-dependent ciliary regulation takes place locally in the vicinity or within the ciliary compartment, almost independently from intracellular Ca^2+ ^concentration [35]. Given that the gradient of free Ca^2+ ^in the cytosol dissipates within 1-2 seconds [36], it appears more likely that cilia form their own compartment where Ca^2+ ^is regulated by active Ca^2+ ^transport in a similar fashion to the intracellular Ca^2+ ^regulatory system. This hypothesis resolves the problem of maintaining physiological levels of intracellular Ca^2+ ^concentration. A number of experimental studies have reported several controversial results relating to the Ca^2+^-dependent mechanism of cilia regulation. For example, it has been reported that spontaneous cilia beat does not require alterations in Ca^2+ ^[31,35], while nucleotide-dependent CBF increase requires Ca^2+ ^[8]. It has also been shown that uncoupling between Ca^2+ ^and CBF can be achieved by inhibition of Ca^2+^-dependent protein calmodulin (CaM) or the cyclic nucleotide pathway [19,32,37].

**Figure 1:**
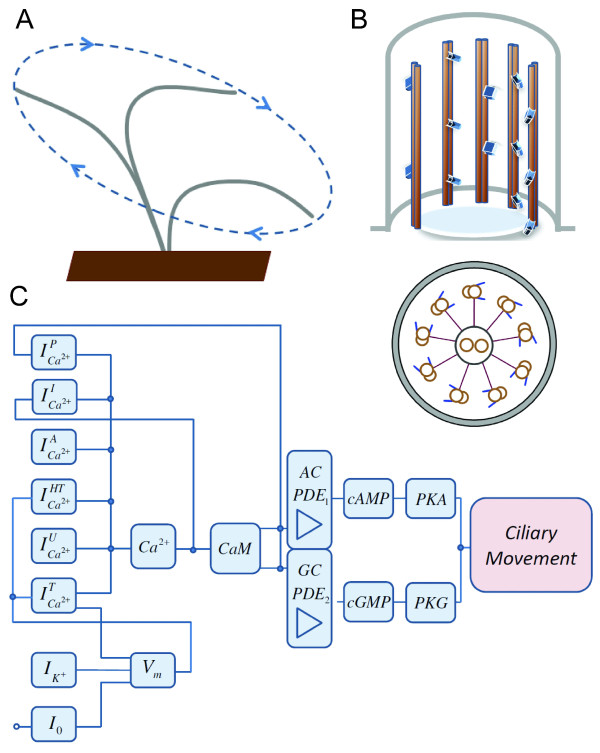
**Schematic diagram of the Ca^2+^-dependent regulation of motile cilium**. (A) Schematic representation of the motile cilium movement trajectory for one complete beating cycle. The cycle is divided into two phases: effective stroke and recovery stroke shown by red and green arrows, respectively. The frequency and the direction of cilia movement is regulated in a highly complex Ca^2+ ^and membrane voltage-mediated manner. (B) A typical cilium consists of an axoneme of nine doublet microtubules. The axoneme is surrounded by a specialized ciliary membrane that is separated from the cell membrane by a zone of transition fibres. This separation creates an intraciliary compartment where key regulatory events take place somewhat independently from the cell body. In particular, intraciliary Ca^2+ ^concentration can significantly differ from intracellular levels. (C) The ciliary motion is regulated by intraciliary Ca^2+ ^levels. The Ca^2+ ^concentration depends on the interplay of ion channels and membrane potential, *V_m_*. The intraciliary Ca^2+ ^concentration is dependent on currents via Ca^2+^, $I_{Ca^{2+}}^{I}$, and K^+ ^channels, $I_{K^{+}}$, the system of active, $I_{Ca^{2+}}^{A}$, and passive, $I_{Ca^{2+}}^{p}$, ions removal, Ca^2+ ^leakage current, $I_{Ca^{2+}}^{U}$, hyperpolarisation-activated currents $I_{Ca^{2+}}^{HT}$, inward current, *I*_0_, and the cilium-to-cell body current, $I_{Ca^{2+}}^{T}$. The conductivities of the channels are modulated by membrane potential. The result of the cross-talk between membrane potential and a variety of channels is that intraciliary Ca^2+ ^can shift between several dynamic modes. The steady-state and dynamic Ca^2+ ^alterations regulate the intraciliary levels of cAMP and cGMP in a Ca^2+^-CaM-dependent manner via the AC, GC and PDE isoforms [65]. Cyclic nucleotides, in turn, define the degree of phosphorylation of dynein filaments in the bases of ciliary axoneme via PKA and PKG kinases. Phosphorylation of dynein filaments regulates the relative doublet microtubules shift and thereby translates to the overall ciliary movement.

These findings suggest that intraciliary Ca^2+ ^does not regulate cilia beat in isolation, but instead does so as part of more complex signalling network. Although it was originally believed that Ca^2+^, cyclic adenosine monophosphate (cAMP) and guanosine monophosphate (cGMP) regulate ciliary beat independently, numerous reports now strongly indicates that all three pathways are tightly interconnected [12,19,32,38-42]. The cAMP-dependent protein kinase (PKA) phosphorylates dynein in the bases of cilia and thereby increases the forward swimming speed in *Paramecium *[43-45]. Similar effects have been reported for PKA-dependent phosphorylation of axonemal targets in mammalian respiratory cilia [46]. Several lines of evidence indicate that PKA and cGMP-dependent kinase (PKG) both phosphorylate specific axonemal targets in a cAMP and cGMP-dependent manner. A schematic diagram for the underlying biochemical machinery for cilia movement regulation is shown in Figure 1C. It is striking that, despite significant experimental characterisation of this system, there is still rather limited mechanistic understanding of how intraciliary Ca^2+ ^and nucleotide interplay relates to CBF.

Another major regulator of ciliary beating is the membrane potential. A number of studies have reported the voltage-dependent effects of ciliary beating. The ciliate *Didinium Nasutum *has been shown to respond both to hyper- and de-polarization of the membrane [47]. The transmembrane potential alterations were shown to be mediated via the potential-dependent Ca^2+ ^channels [48]. Electrophysiological studies in *Paramecium caudatum *have revealed complex relationships between ciliary Ca^2+ ^currents, intraciliary Ca^2+ ^concentration and transmembrane potential in the regulation of ciliary motility [49-55].

A number of previous computational studies have analysed various aspects of cilia movement regulation. One earlier model assessed the degree of synchronization between small ciliary areas [56]. The effects of viscosity have been investigated in mucus propelling cilia in [57]. The authors found that increasing the viscosity not only decreases CBF, but also changes the degree of correlation and synchronization between cilia. The mechanical properties of cilia motion were studied in an attempt to understand the ciliary dynamics in [58]. The authors concluded that bending and twisting properties of the cilium can determine self-organized beating patterns. While these reports offer valuable insights into the regulatory mechanisms of cilia, a number of essential questions remain unresolved. For example, there has not been a detailed analysis of how individual Ca^2+ ^currents influence intraciliary Ca^2+ ^levels. It also remains unclear how Ca^2+ ^modulates nucleotide levels and membrane potential, and how such regulation affects ciliary movement. None of these reports have elucidated the underlying mechanisms governing the interplay between intraciliary Ca^2+ ^and nucleotide alterations and CBF.

In this study, we integrate the available experimental information on the molecular pathways that regulate intraciliary Ca^2+ ^concentration into a comprehensive mathematical model. By applying systems analysis, we elucidate the mechanisms of intraciliary Ca^2+ ^spike generation, analyse the properties of such spikes and demonstrate the conditions under which the Ca^2+ ^surges can become repetitive. We carry out detailed investigations of the individual current contributions to the regulation of the intraciliary Ca^2+ ^concentrations and elucidate both steady-state and dynamic responses of Ca^2+ ^currents and intraciliary Ca^2+ ^concentration dynamics in response to the altered transmembrane potential shift. The model allows detailed elucidation of transmembrane potential and intraciliary Ca^2+ ^coupling.

We employ the proposed model in order to understand the underlying molecular mechanisms of the crosstalk between Ca^2+^, membrane potential and nucleotide pathways that regulate ciliary movement. The systems model allows detailed analysis of the individual current contributions to the intraciliary homeostatic Ca^2+ ^levels. Furthermore, we establish specific regulatory mechanisms for Ca^2+ ^and cyclic nucleotide-dependent cilia movement characteristics. Crucially, our model predicts the possibility of several ciliary beating modes and describes specific conditions that initiate them. Specifically, we describe intraciliary Ca^2+ ^dynamic modes that regulate healthy and pathologic cilia beating. We use these findings in order to propose experimentally testable hypotheses for possible therapeutic interventions in human diseases associated with pathologic cilia motility.

## Results

### A new model for the interplay between Ca^2+ ^and K^+ ^currents and transmembrane potential alterations

A new model for the regulation of ciliary movement that combines multiple Ca^2+ ^and K^+ ^currents [59-62] and transmembrane potential has been developed. In this model, the intraciliary Ca^2+ ^levels are modulated by Ca^2+ ^currents through the channels of passive and active Ca^2+ ^transport, the current from the cilium into the cell body, the Ca^2+ ^leakage current, and depolarisation and hyperpolarisation-activated currents. Variable extracellular conditions have continuous impact on the transmembrane potential which is intertwined with transmembrane ion currents and intraciliary Ca^2+ ^homeostasis.

The overall network that regulates ciliary movement is divided into several functional modules (Figure 1C). One module combines all Ca^2+ ^and K^+ ^currents that define intraciliary Ca^2+ ^homeostasis and the transmembrane potential. One of the most essential intraciliary Ca^2+ ^binding proteins, CaM [63,64], selectively regulates the activities of adenylate cyclase (AC), guanylate cyclase (GC) and phosphodiesterases (PDE), and thereby modulates the intraciliary levels of adenosine monophosphate (cAMP) and guanosine monophosphate (cGMP) in a Ca^2+ ^dependent manner [65]. The cAMP- and cGMP-dependent kinases phosphorylate dynein proteins in the bases of cilia and thereby induce the mechanical cilia movement. The complete set of equations making up the proposed model is presented in the Methods section. Below we provide a number of new insights into the mechanism of cilia regulation via a detailed investigation of the properties of this model.

### The mechanism of Ca^2+^-dependent inhibition of Ca^2+ ^channels

A subset of intraciliary Ca^2+ ^channels have been reported to operate in an intraciliary Ca^2+ ^dependent manner and have been proposed as major regulators of ciliary beat [49-51]. It is established that Ca^2+ ^current is not inhibited by the double pulse application of depolarization impulses under voltage clamp conditions in those situations when the first transmembrane potential shift is equal to the equilibrium Ca^2+ ^potential (+120 mV) [66]. Further experimental evidence reveals that Ca^2+ ^current inactivation kinetics are delayed when Ca^2+ ^ions are partially replaced by Ba^2+ ^ions [67-71]. Altogether these findings suggest that the channels are not inhibited directly by the depolarizing shift of transmembrane potential, but that instead their conductivity is dependent on the intraciliary Ca^2+ ^concentration. Some decrease of the inward current amplitude (by approximately 25%) upon transmembrane potential shift into the Ca^2+ ^equilibrium level can be explained by the fact that K^+ ^currents can contribute to the overall current measurements. Here we consider the intraciliary Ca^2+ ^concentration-dependent Ca^2+ ^channel inhibition and employ the developed model to analyse two potential scenarios for the Ca^2+ ^channel conductivity regulation. In one case, Ca^2+ ^ions bind to the Ca^2+ ^binding site on the channel and thereby inhibit the channel's conductivity by direct interaction. The other possibility is that the Ca^2+ ^binding protein interacts with the Ca^2+ ^ion first and then this complex binds to the channel and inhibits its conductivity. In both cases the conductivity dependence on transmembrane potential is assumed to be monotonic according to the experimental data [66].

### Direct Ca^2+^-dependent Ca^2+ ^channel conductivity inhibition

**Figure 2:**
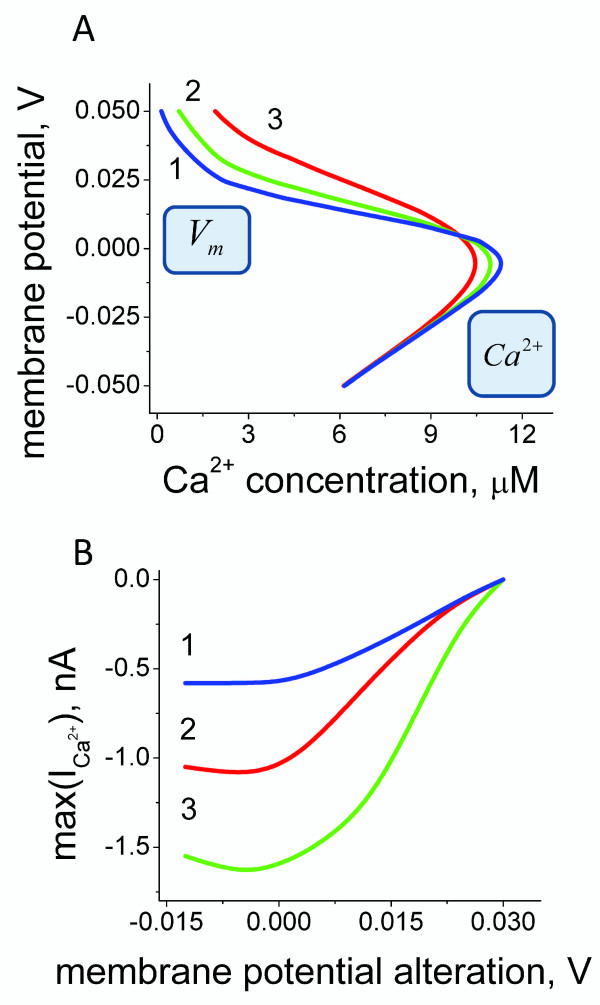
**Static and dynamic intraciliary Ca^2+ ^concentration levels under membrane potential fixed conditions**. (A) The model predictions for the relationship between the membrane potential and intraciliary Ca^2+ ^concentration. (B) The maximum amplitude of Ca^2+ ^current generated in response to the transmembrane potential shift is shown as function of the applied shift. The model predictions are obtained by setting the parameter α to 2, 3, and 4 in equations 18-21. The parameter α reflects the steepness of Ca^2+ ^current dependence on membrane voltage. The model-based analysis shown here unravels the bell-shaped dependence of intraciliary Ca^2+ ^concentration on membrane potential and the inverse relationship between the Ca^2+ ^current magnitudes and the degree of membrane depolarisation.

We first investigate a potential intraciliary Ca^2+ ^regulatory mechanism via direct Ca^2+ ^ion binding-dependent Ca^2+ ^channel inhibition. The relationship between the Ca^2+ ^channel conductivity and the transmembrane potential has been experimentally characterized by an early study in *Paramecium *species [66]. The equation (18) in the Methods section approximates the experimentally established dependence. In the case of direct Ca^2+^-dependent Ca^2+ ^channel inhibition, one can show that the nullclines for non-dimensional Ca^2+ ^concentration ($\frac{dCa^{2+}}{dt}=0$) and for the number of open channels ($\frac{dn}{dt}=0$) from the system of differential equations (20) intersect at one stable point for all values in the physiological range of model parameters. The numerical solutions of the coupled differential equations (20) allow us to obtain the solutions for how the steady-state Ca^2+ ^levels depend on the transmembrane potential. The model predictions for the steady-state ciliary Ca^2+ ^channel conductivity dependence on the transmembrane potential under the voltage clamp conditions are shown on Figure 2A. The extracellular conditions are subject to constant change both in the case of ciliates as well as for multicellular organisms. The modifications in the external environment continuously shift the transmembrane potential. In order to estimate how the transmembrane potential alterations affect the inward Ca^2+ ^current we derived the dependence for the Ca^2+ ^ion flow (equation (21) in the Methods section). The experimental data-based (Figure 2A) [66] model for the inward Ca^2+ ^current dependence on membrane potential (Figure 2B) predicts a significant reduction of the inward Ca^2+ ^current amplitude as a function of membrane depolarization.

**Figure 3:**
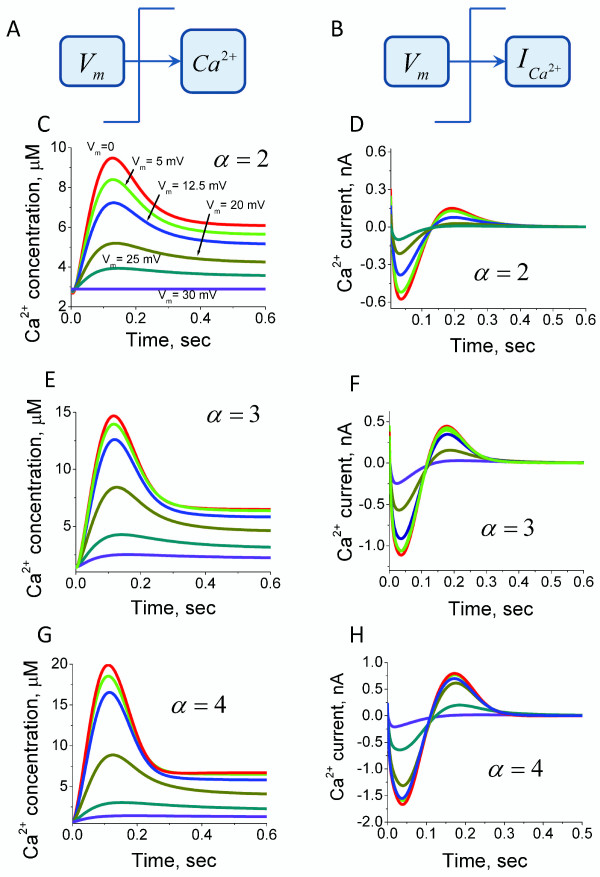
**Systems model predictions for intraciliary Ca^2+ ^concentration and current responses to the transmembrane potential shift**. The intraciliary Ca^2+ ^concentrations (A) and Ca^2+ ^currents (B) are calculated according to the model with direct Ca^2+^-mediated ciliary Ca^2+ ^channels inhibition in response to variable degree of transmembrane potential shift. The responses are colour coded according to the degree of applied transmembrane potential shift. The violet and red coloured lines represent dynamic responses obtained after the smallest and the largest depolarising shift of membrane potential, respectively. The non dimensional membrane potential values following voltage shift from initial *ψ*_0 _= -1.2 are shown in (C) and remain the same throughout the figure. The calculations suggest that ciliary membrane depolarisation induces an intraciliary Ca^2+ ^spike over a wide physiological range of depolarising conditions (C, E and G), whereas the current generates a biphasic response (D, F and H). The calculations were carried out for three different levels of parameter *α*, which reflects the steepness of the Ca^2+ ^channels conductivity dependence on membrane potential.

We next investigated the dynamic alterations of the intraciliary Ca^2+ ^concentrations and the inward Ca^2+ ^current in response to the transmembrane potential shifts. Equations (20) and (21) in the Methods section were used for quantitative estimations of the Ca^2+ ^concentration and current responses to the normalised membrane potential shifts. The model predicts that the rapid depolarising alterations of the ciliary transmembrane potential leads to the generation of single Ca^2+ ^spikes (Figure 3). Such responses can take place when Ca^2+ ^channels are inhibited by Ca^2+ ^ions and the channel's conductivity depends on the membrane potential in a monotonic manner (equation 18). The amplitude of those impulses depends on the steepness of the Ca^2+ ^channels conductivity dependence on the membrane potential. The mechanism of the Ca^2+ ^spike generation is mainly due to the delay of the Ca^2+^-induced inhibition with respect to the Ca^2+ ^conductivity alteration characteristic times. The described mechanism of the Ca^2+ ^spike generation will only work if the Ca^2+ ^currents significantly alter the Ca^2+ ^concentration in the intraciliary compartments.

### Indirect Ca^2+ ^channel conductivity regulation

In the previous section we considered Ca^2+^-dependent Ca^2+ ^channel regulation under the assumptions that Ca^2+ ^channels have an intracellular Ca^2+ ^binding site and Ca^2+ ^ion binding closes the channels. However, several experimental studies have suggested that the conductivity of Ca^2+ ^channels in cilia can also be regulated indirectly, via a Ca^2+ ^binding protein. At present, there is no direct experimental evidence that explicitly favours either direct or indirect regulatory mechanism. We, therefore, investigated the second possibility for indirect Ca^2+^-dependent Ca^2+ ^channels conductivity inhibition.

**Figure 4:**
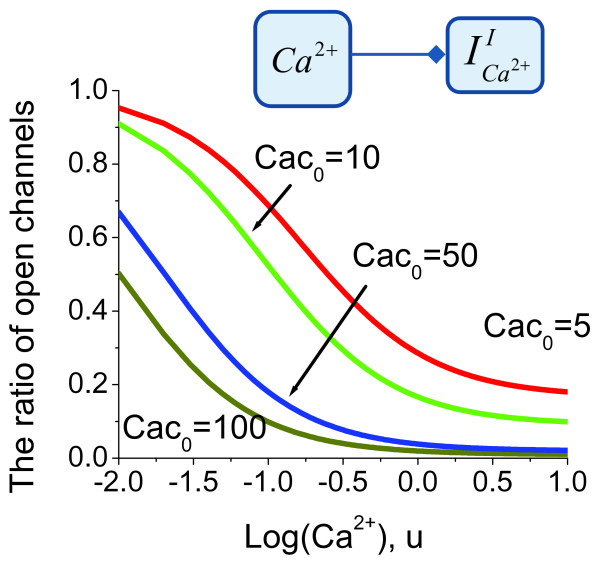
**The predicted probabilities for Ca^2+ ^channels to be in the open state**. The indirect Ca^2+^-mediated inhibition of Ca^2+ ^channels via a Ca^2+^-sensor protein, *Cac*_0_, is shown for a range of Ca^2+^-binding protein concentrations. The higher levels of Ca^2+^-sensor contributes to a greater degree of inhibition, although the general mechanism remains the same.

The model for Ca^2+ ^ion interactions followed by the interactions with the Ca^2+ ^channels (described in the Methods section) is developed in line with a previously suggested modelling methodology for Ca^2+^-CaM interactions [63,64]. The model predictions for the number of open channels as a function of intraciliary Ca^2+ ^concentration are shown on Figure 4 as derived by equation (25) in the Methods section. Given that the exact nature of the Ca^2+ ^binding protein acting as a mediator between Ca^2+ ^ions and Ca^2+ ^channels is not established, equation (25) has been solved for different physiologically possible ratios of total number of channels to the dissociation constant for the Ca^2+^- binding protein interactions with Ca^2+ ^channels. According to the derived models, the comparison of direct versus indirect Ca^2+ ^channels inhibition can be carried out by setting the non dimensional Ca^2+ ^concentration to zero (*u *= 0 in equation (25)). In this case, the predictions of equation (25) for indirect Ca^2+ ^channels conductivity inhibition almost coincide with the model for direct Ca^2+^-dependent inhibition. In both models most of the Ca^2+ ^channels are open in the lower range of Ca^2+ ^concentrations. However, the model for indirect Ca^2+ ^channels inhibition predicts that the number of open Ca^2+ ^channels would equal $\frac{1}{\text{ca}{\text{c}}_{0}+\text{1}}$ when intraciliary Ca^2+ ^reaches high concentrations. In other words, in the case of the indirect mechanism of inhibition, high Ca^2+ ^does not inhibit the channels completely. The number of remaining channels in the open state would depend on the concentration of the regulatory Ca^2+ ^binding protein.

**Figure 5:**
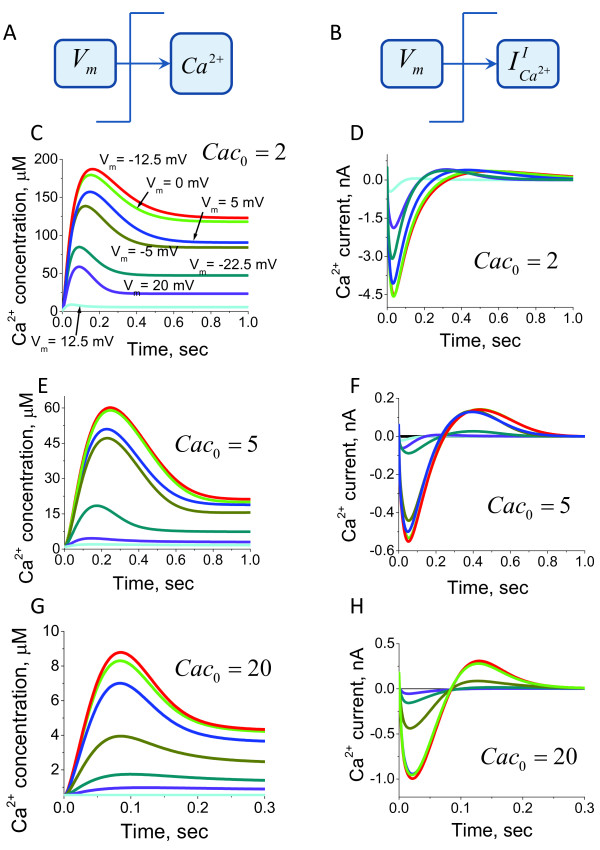
**The responses of Ca^2+ ^current and concentration to the potential shift via indirect Ca^2+ ^inhibition**. The intraciliary Ca^2+ ^concentration (A) and Ca^2+ ^current (B) responses to membrane depolarisation are computed under the assumption of indirect Ca^2+ ^channel inhibition for different concentrations of Ca^2+^-sensor protein, *Cac*_0_. The model predicts that in response to fast membrane depolarisation, the ciliary coupled Ca^2+ ^and membrane potential system generates an intraciliary Ca^2+ ^concentration spike similar to the one observed under the direct Ca^2+^-inhibition assumptions. The non dimensional membrane potential values following voltage shift from the initial *ψ*_0 _= -1.2 are shown in (C) and are the same throughout the figure. The major difference between direct (Figure 3) and indirect mechanisms of inhibition occurs in the steady-state levels of Ca^2+ ^concentration that takes place after the transitional process. The comparison of intraciliary Ca^2+ ^concentration responses for increasing levels of the Ca^2+ ^sensor protein (C, E and G) predicts that intraciliary Ca^2+ ^concentrations following the voltage shift are inversely dependent on the Ca^2+ ^sensor concentration, *Cac*_0_. Different concentrations of Ca^2+ ^sensor protein do not affect the membrane depolarisation-induced Ca^2+ ^current (D, F and H).

**Figure 6:**
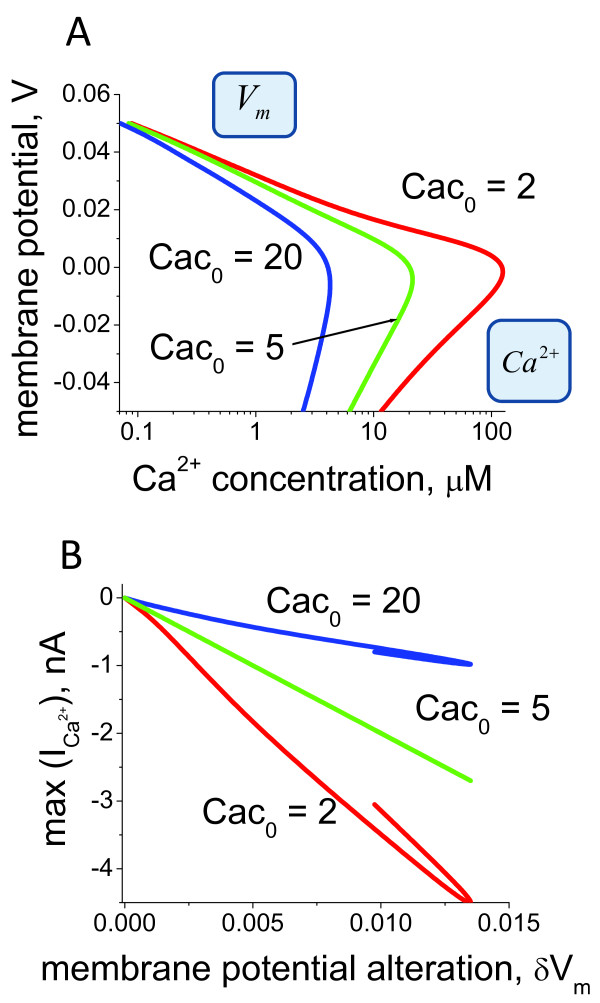
**The membrane potential effects on Ca^2+ ^current under fixed voltage**. (A) The relationship between membrane potential and intraciliary Ca^2+ ^concentration is shown under voltage clamp conditions. (B) The inward current amplitude dependence as a function of a fast shift in the holding membrane potential. Calculations were performed for different concentrations of Ca^2+ ^sensor protein, CaC_0_. The model predicts qualitatively similar responses for a relatively wide range of Cac_0 _concentrations.

We next analysed the dynamics of the ciliary Ca^2+ ^channels inhibition in response to a change in intraciliary Ca^2+ ^concentration and in the transmembrane potential. As mentioned earlier, the Ca^2+ ^channel conductivity is not inhibited by the membrane potential, but rather has a monotonic dependence on the transmembrane potential difference as shown on Figure 2B. We found that the characteristic time $\tau_{Ca^{2+}}$ of Ca^2+ ^channel alterations in response to step changes in Ca^2+ ^is inversely proportional to the total number of channels. The model predictions for intraciliary Ca^2+ ^concentration and inward Ca^2+ ^current in response to depolarising transmembrane potential changes from *V*_0 _to *V*_1_, are shown on Figure 5. According to the derived equation (30) in the Methods section, the inward Ca^2+ ^current changes with a characteristic time τ*_V_*, which can only be estimated by considering the K^+ ^current contribution. In the first instance we only consider active and passive Ca^2+ ^transport (equation (31) in Methods). We neglected by the kinetics for the channels of active transport due to the assumption that the kinetics of alterations of active transport are much faster that the characteristic alteration times of passive transport (equation (32) for the Ca^2+ ^currents). The model predictions for the steady-state dependence of transmembrane potential on intraciliary Ca^2+ ^concentration and the dynamic Ca^2+ ^current amplitude dependence on the membrane potential are shown in Figure 6A and 6B, respectively.

**Figure 7:**
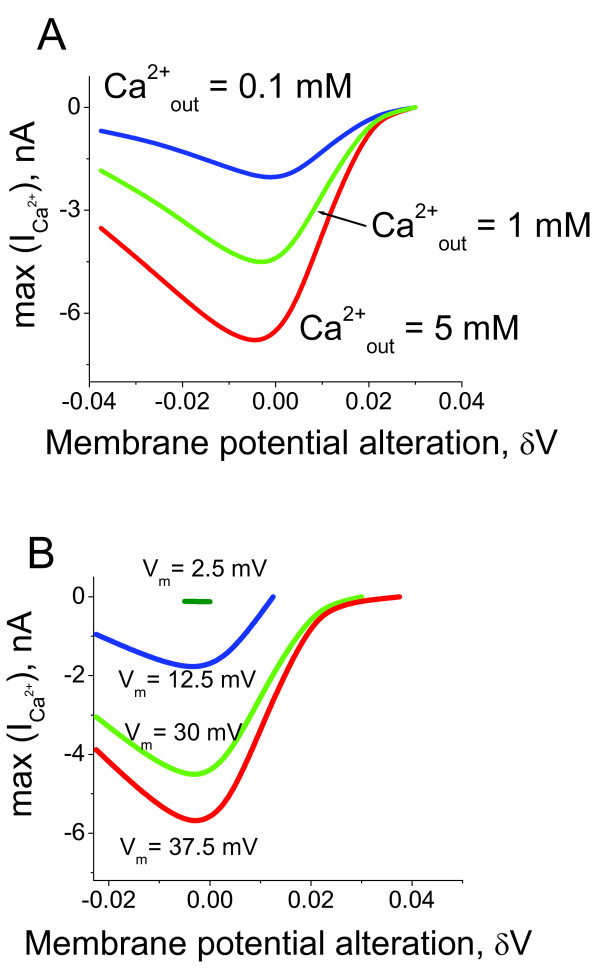
**The model predictions for Ca^2+ ^current in response to membrane potential shift**. The model predictions for Ca^2+ ^current amplitude shown as a function of extracellular Ca^2+ ^concentration and the initial membrane potential values before the voltage shift was applied. (A) The dependence of Ca^2+ ^current amplitude on the transmembrane potential alterations is calculated for different extracellular Ca^2+ ^concentrations. (B) The graph shows the Ca^2+ ^current amplitude dependence on the initial values of the potential when the depolarising voltage shift was applied. The model shows that the ciliary system properties are reasonably conserved under significant variations of extracellular Ca^2+ ^and over a wide range of membrane potential values.

The cilia's external environment is subject to constant change and can significantly affect the behavioural responses of ciliates and modulate ciliary beating in multicellular organisms. In order to account for the effects of extraciliary Ca^2+ ^variations we estimated the amplitudes of intraciliary Ca^2+ ^spike generation under different external Ca^2+ ^concentrations and variable transmembrane potentials. Figure 7A shows that the increase of the Ca^2+ ^concentration in the external solution increases the amplitude of the generated intraciliary Ca^2+ ^spike. The amplitude of the spikes goes to zero when the membrane potential equals the equilibrium potential for Ca^2+ ^ions. The increase of membrane potential decreases the amplitude of the Ca^2+ ^current (Figure 7B). This effect is due to the increase of the steady-state intracilia Ca^2+ ^level and Ca^2+^-dependent inhibition of the Ca^2+ ^channels.

We noted earlier that there is a Ca^2+ ^current in the cilia which transfers ions from the cilia into the cellular compartments. This current can be described by equation (11) in Methods. The contribution of cilium-to-cell body current to the intraciliary Ca^2+ ^concentration dynamics was evaluated experimentally in [72,73]. It was shown that under depolarized membrane potential conditions the contribution of this current is very small and the intraciliary Ca^2+ ^is mainly pumped out of the cilia into the extracellular space by the active Ca^2+ ^transport. According to other observations, Ca^2+ ^current from cilia into the cellular compartment can be larger than the current generated by the active Ca^2+ ^transport. In order to investigate the role and contribution of the cilia-to-cell compartment current, we introduced its contribution to the intraciliary Ca^2+ ^concentration dynamics (equation (34)). We performed qualitative analysis of the Ca^2+ ^concentration alterations in the cilia in the presence of the cilium-to-cell current and compared the Ca^2+ ^dynamics with the case when this current was not present. We found that although the cilium-to-cell body current influences the intraciliary Ca^2+ ^concentration levels, it does not change the dynamics qualitatively when the membrane potential is depolarized and fixed.

Our findings suggest that the cilium represents an excitable system with unique properties. The Ca^2+^-dependent inhibition of Ca^2+ ^channels inhibition allows for the generation of single impulses of variable amplitude proportional to the degree of membrane depolarisation caused by variations in the external concentrations of ions. This system is able to generate a single spike despite unpredictable variations of ionic concentrations in the environment and is, therefore, very robust to alterations in the external conditions. Another interesting aspect of the ciliary excitation is the ability of the system to generate regulatory intraciliary Ca^2+ ^impulses proportional to the degree of membrane depolarisation (Figures 3 and 5). This property can allow cells to sense and "automatically" respond to alterations in their environment.

### The contribution of K^+ ^currents

In the previous section, we analysed the dynamic properties of the intraciliary Ca^2+ ^system under voltage clamp conditions. Several lines of evidence suggest that K^+ ^currents contribute to the currents registered in cilia under voltage clamped conditions. The existence of K^+ ^currents in cilia is supported by a number of experimental studies. The experimental data shows that the measured current is not equal to zero when the membrane potential equals the equilibrium membrane potential for Ca^2+ ^ions. Instead, the current equals zero when membrane potential is about 10 mV while the equilibrium potential for Ca^2+ ^ions equals 120 mV [66]. This observation suggests that both Ca^2+ ^and K^+ ^currents contribute to the overall current measured at early stages of current registration under voltage clamp, and therefore both currents need to be taken into the consideration in order to advance understanding of the mechanisms involved in ciliary regulation. At the same time, it has so far been impossible to register Ca^2+ ^currents by inhibiting the K^+ ^contribution. Various compounds can only partially block the K^+ ^current when applied from inside of the membrane. Ciliary K^+ ^currents have also been measured separately from Ca^2+ ^currents.

**Figure 8:**
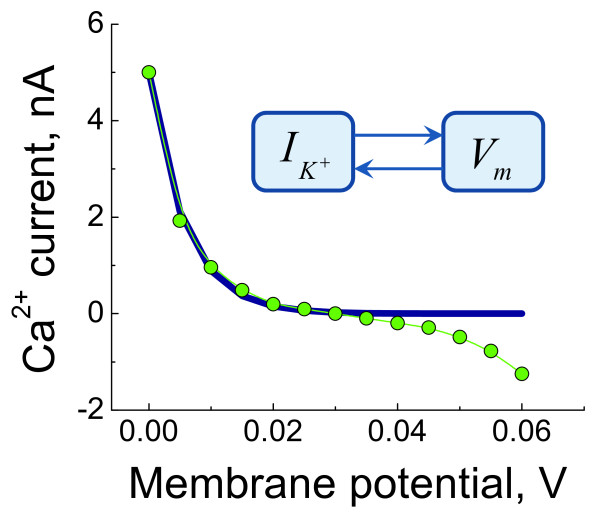
**Current-voltage characteristic for the steady-state K^+ ^current**. The model predictions for the K^+ ^current as a function of membrane potential are compared with the experimentally measured voltage-current. The model elicits accurate agreement with the experimental data for the physiologically relevant range of membrane potential alterations.

**Figure 9:**
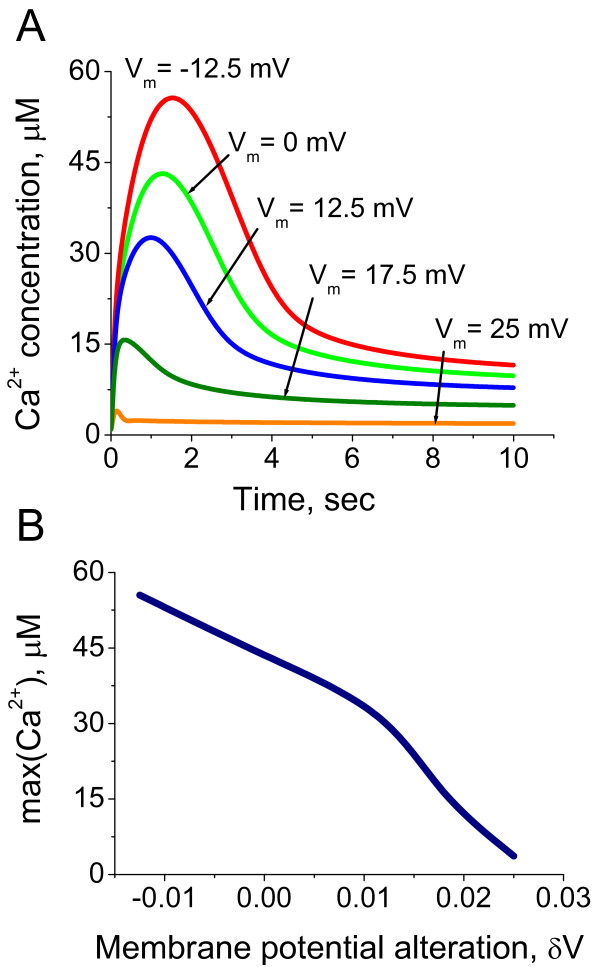
**Ca^2+ ^concentration dynamics response to transmembrane potential shift in the presence of K^+ ^current**. (A) The intraciliary Ca^2+ ^dynamics is computed in response to transmembrane potential shift by incorporating the contribution from K^+ ^channels to the overall current. Comparison of these results with Figures 3 and 5 suggests that while K^+ ^currents introduce some quantitative changes, the overall qualitative characteristics of the response remain the same. The non dimensional membrane potential values following voltage shift are indicated for each predicted response. (B) The amplitude of the generated Ca^2+ ^concentration response is shown as a function of membrane potential alteration. The amplitude of the current is approximately linearly inversely proportional to the magnitude of the applied membrane potential shift.

In order to account for the contribution of K^+ ^currents to the regulation of intraciliary Ca^2+ ^concentration, we developed a model for the regulation of K^+ ^currents by membrane potential (equation (35) in Methods). The dependence of K^+ ^conductivity on the membrane potential is described by equation (36). Figure 8 shows the measured experimental values and the approximating curve calculated according to equation (36). The current on Figure 8 is normalized to 1 when membrane potential equals 0. In order to approximate the current, we only used the experimental values obtained under the membrane depolarized conditions, and this experimental data is approximated by equation (36). The predictions for intraciliary Ca^2+ ^dynamics and Ca^2+ ^impulse amplitude in response to the shift in membrane potential are shown in Figure 9A and 9B, respectively. A comparison of the membrane potential shift-induced intraciliary Ca^2+ ^spike generation in the presence of the K^+ ^currents (Figure 9A) with the model when K^+ ^channels are not incorporated (Figure 3 and 5), suggests that while the K^+ ^currents change the quantitative values of the Ca^2+ ^concentration dynamics, the general shape of the Ca^2+ ^response remains the same.

### The transmembrane potential dynamics in the absence of voltage clamp

In the previous sections we investigated the mechanisms of the transmembrane potential shift-dependent Ca^2+ ^spike generation under voltage clamp conditions. However, Ca^2+ ^currents themselves can alter the membrane potential. Here we incorporate the membrane potential dependence on Ca^2+ ^currents and investigate the membrane potential dynamics in the absence of voltage clamp (equations (40) and (41)). The non dimensional Ca^2+ ^concentration and membrane potential are described by equation (42).

**Figure 10:**
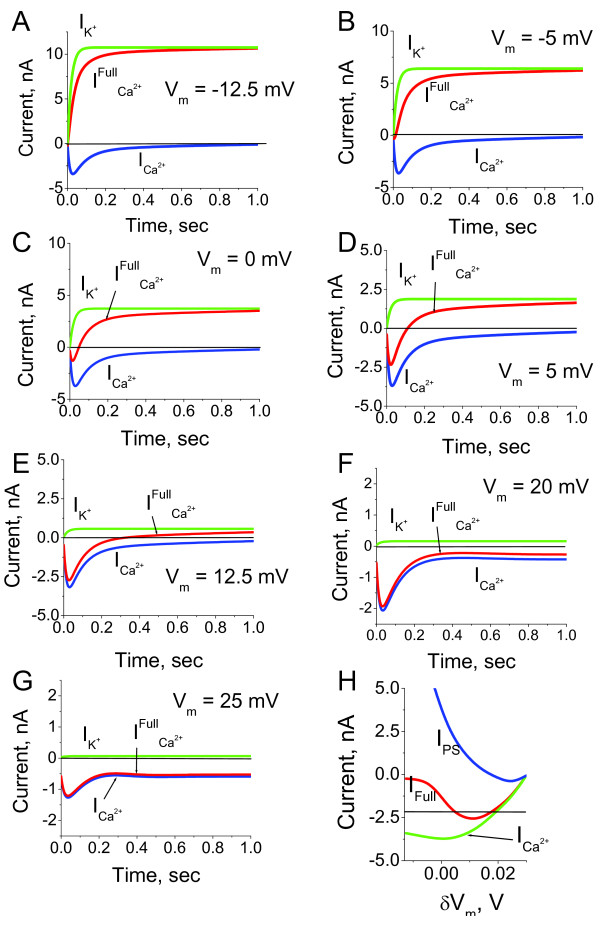
**The dynamics of current alterations in the absence of voltage clamp**. The Ca^2+ ^and K^+ ^current dynamics were calculated in response to the membrane potential shift in the absence of voltage holding conditions (A-G). The membrane potential was depolarised from the normalised value *V*_0 _= -30 mV to -12.5 mV (A) and 25 mV (G), which represent the smallest and the largest change shift, respectively. The currents elicited by the membrane potential alteration within the range are shown in (B)-(F). $I_{Ca^{2+}}$, $I_{K^{+}}$ and *I_Full _*represent calcium, potassium and full currents, respectively. The comparison with the Ca^2+ ^current responses to membrane potential shift obtained under the voltage clamped conditions (Figure 3, 5 and 9) reveals significant differences in the dynamics. (H) Voltage current characteristic is calculated using the current amplitudes from the currents dynamics for the full (*I_Full_*) and Ca^2+ ^($I_{Ca^{2+}}$) currents. The steady-state voltage current relationship is calculated according to the stationary current values, (*I_PS_*).

The predictions for the individual Ca^2+ ^and K^+ ^current responses to various membrane potential shifts are shown in Figure 10. One can note that the dynamics of the responses significantly differs between the ones obtained under voltage clamp conditions and the situation when the membrane potential is not fixed, but dependent on the currents (Figures 3, 5 and 9). Voltage current characteristics for Ca^2+^, K^+ ^and full currents calculated using the current amplitudes from the currents dynamics are shown on Figure 10H. One can note that the current is an almost exponentially growing function of membrane potential.

The monotonic dependence of Ca^2+ ^current on transmembrane potential, and simultaneous Ca^2+^-dependent inhibition of Ca^2+ ^channels, represents a classical problem of two interconnected variables: intraciliary Ca^2+ ^and membrane potential. In this system, increasing Ca^2+ ^current with transmembrane potential depolarisation represents a positive feedback loop mechanism, whereas the intraciliary Ca^2+ ^concentration-dependent Ca^2+ ^channels inhibition represents a negative feedback loop. We, therefore, sought to investigate the range of potential dynamical properties of the ciliary system emerging from the coupling of Ca^2+ ^current and membrane potential described by equations (42).

**Figure 11:**
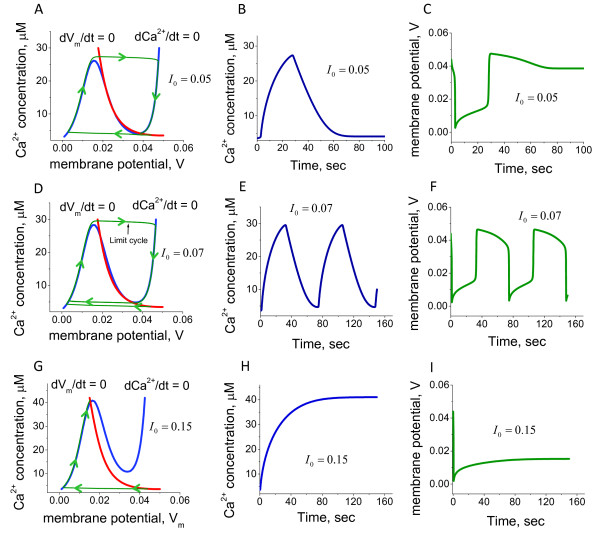
**The model predictions for coupled intraciliary Ca^2+ ^concentration and membrane potential alterations**. The systems model for intraciliary Ca^2+ ^concentrations, Ca^2+ ^and K^+ ^currents coupled with membrane potential responds to applied inward current in a dynamically diverse fashion. The dynamic mode of the ciliary system is defined by the intersection of nullclines shown on phase diagrams for three representative values of inward current, *I*_0_. A. A small inward current, *I*_0_, initiates the generation of a small pulse of intraciliary Ca^2+ ^(B) and membrane potential (C). D. A larger inward current can shift the system into the mode of sustained oscillations of both intraciliary Ca^2+ ^(E) and membrane potential (F). G. Significantly higher values of inward current shift the intersection of the null clines to a higher region with a stable solution. This leads to a switch type of response to a higher steady-state level of Ca^2+ ^(H) and membrane potential (I). The model describes a novel mechanism of ciliary excitability and predicts that the ciliary system can generate either a single impulse, generate sustainable oscillations, or operate as a switch between lower and higher Ca^2+ ^and membrane potential levels.

Figure 11 shows the Ca^2+ ^current and membrane potential dynamics and the phase diagrams for an increasing range of inward current. We found that the inward current into the cilium can modify the dynamic properties of the Ca^2+^-membrane potential system. In all cases, the null cline $\frac{dV}{dt}=0$ represents the N-shaped curve. In the physiological range of non-dimensional membrane potential *V *(50 mV, -0.025 mV) and intraciliary Ca^2+ ^(*u*) from 0.04 to 40 *μM*, the null cline $\frac{dCa^{2+}}{dt}=0$ shows a monotonic growth.

One can clearly see that there is significantly different response for different values of the inward current. When the influx of the ions is relatively small, the $\frac{dCa^{2+}}{dt}=0$ null cline intersects the $\frac{dV}{dt}$ = 0 null cline in the left descending area (Figure 11A); such a null cline crossing results in a stable solution. In this case the system responds by the generation of a single impulse of both intraciliary Ca^2+ ^concentration and the membrane potential followed by a return to homeostatic levels (Figure 11A, B and 11C). Further increasing the current causes the null cline $\frac{dCa^{2+}}{dt}=0$ to intersect with the null cline $\frac{dV}{dt}=0$ in the middle region of the ascending area, leading to an unstable solution with a limit cycle formed around the area that represents the oscillations. (Figure 11D, E and 11F). However, further increase of the current causes the $\frac{dCa^{2+}}{dt}=0$ null cline to intersect with the null cline $\frac{dV}{dt}=0$ in the right descending area, resulting in a stable solution with a slight increase of the homeostatic Ca^2+ ^and membrane potential levels (Figure 11G, H and 11I). The key conclusion from this analysis is that the external ionic conditions can initiate essentially different dynamic properties of the system regulating ciliary movement. One of the key factors that affect the ciliary beat cycle is the level of intraciliary Ca^2+^. Our findings suggest that in response to the external conditions, there are several possibilities for intraciliary Ca^2+ ^upregulation. The system can generate a single spike (Figure 11B) of variable amplitude (data not shown), permanently increase Ca^2+ ^in a dynamic fashion and maintain the high intraciliary levels (Figure 11E), or operate in a monostable multivibrator mode (cilia can generate a Ca^2+ ^spike in response to any alteration of membrane potential) (Figure 11H). These three possibilities can be associated with the different modes of ciliary beat observed in human cilia as well as in various ciliates.

The dynamic properties of excitable systems with two interdependent variables are reasonably well understood at a theoretical level. In the present case, Ca^2+ ^and membrane potential represent the slow and fast variables, respectively. This study, therefore, establishes that the dynamic properties of ciliary systems, where the Ca^2+ ^and K^+ ^channel conductivities represent monotonic function of membrane potential and the Ca^2+ ^channels conductivity inversely depends on intraciliary Ca^2+ ^concentration, are comparable with the properties of excitable systems based on the "N-shape" dependence of the Na^2+ ^channel conductivity on membrane potential [74]. At the same time, it is essential to note that the mechanism of excitation described in motile cilia is different from the "classical" one described in most excitable cells and systems that involve IP_3 _Ca^2+ ^channels [75,76].

### The membrane hyperpolarisation-dependent currents modulate the excitatory properties of the ciliary system

The ciliary transmembrane potential can shift in two directions. In the previous section we investigated the intraciliary Ca^2+ ^responses caused by membrane depolarisation. Here we assess the implications of the membrane hyperpolarisation which has been shown to activate the current from cilia into the cell body [77,78]. We introduced the corresponding term into our model for the Ca^2+ ^ions movement via the membrane as a function of the corresponding membrane potential shift (equation 43). By assuming the potential independent mechanism for Ca^2+ ^and K^+ ^ion expulsion, the system of intraciliary Ca^2+ ^and membrane potential is derived as shown in equation (46) in the Methods section.

**Figure 12:**
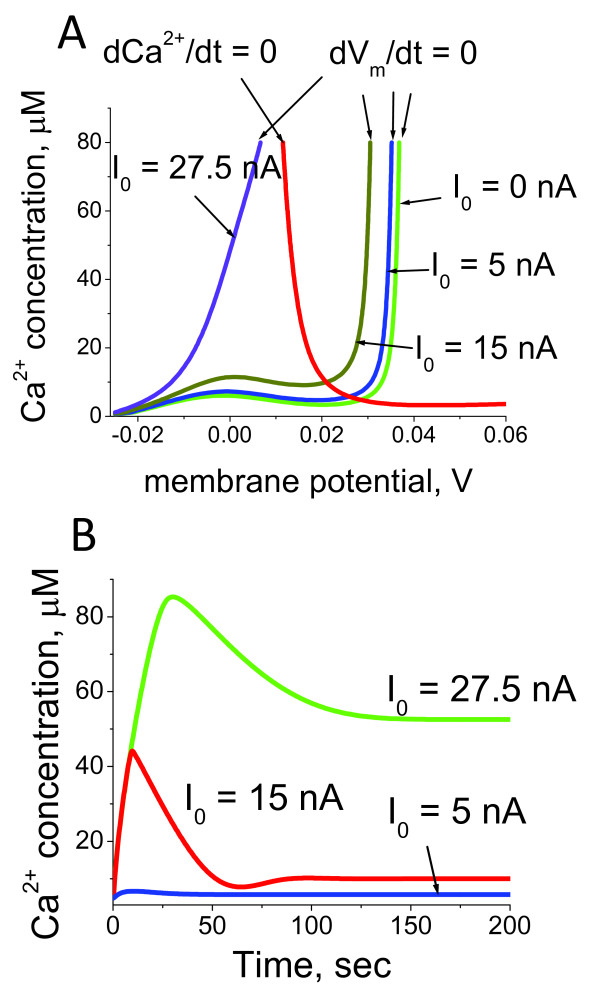
**The "monostable multivibrator" mode occurs in response to membrane hyperpolarisation**. The model predictions are shown for the ciliary response to membrane hyperpolarisation. The phase diagram analysis (A) suggests that the nullclines for Ca^2+ ^and membrane potential *V *always intersect at a single point that correspond to a steady-state solution in the whole physiological range of possible inward current values, *I*_0_. The model shows that this type of system always generates a Ca^2+ ^impulse in response to any alteration of inward current, *I*_0_, within the physiological range (B).

**Figure 13:**
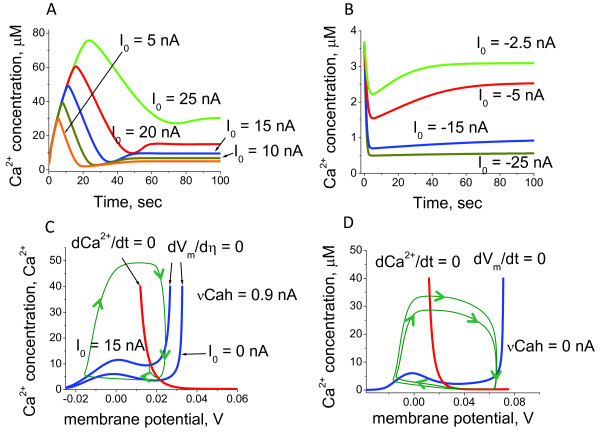
**The hyperpolarisation-mediated Ca^2+ ^spike is proportional to inward current**. The effects of hyperpolarisation-mediated currents are described as a function of inward current magnitude. The computational calculations predict that in the presence of hyperpolarisation induced currents, the ciliary system always generates Ca^2+ ^impulses proportional to the applied inward current in both positive (A) and negative (B) ranges of values. The comparison of phase diagrams in the presence (C) and absence (D) of the hyperpolarisation-induced current *vCah *suggests that the Ca^2+ ^spike generation in response to inward current alteration is due to the hyperpolarisation-mediated currents. In the absence of hyperpolarisation the solution becomes unstable with a limit cycle and the system undergoes sustained oscillations (D).

The model predictions for intraciliary Ca^2+ ^and membrane potential dynamics in the absence of the hyperpolarisation-induced current are shown in Figure 11D, E and 11F. In this case the system remains in the mode of steady oscillations. We found that such oscillatory mode can be significantly modulated by the hyperpolarisation-induced and the external conditions-dependent current. Figure 12A shows the phase diagram in the presence of the hyperpolarising current. In contrast to the situation described on Figure 11 when the hyperpolarising current was absent, the ciliary system generates a single spike in response to alteration of external ionic concentrations over the whole physiological range of the inward current (Figure 12B and 12C). Under certain combinations of the hyperpolarisation-induced currents and the Ca^2+^-dependent K^+ ^currents the phase diagram modifies in a manner so that the system responds by generating a single spike in response to alteration of the inward current of any magnitude (Figure 13). This indicates that the ciliary system under membrane hyperpolarising conditions can become a monostable multivibrator.

### The role of cilia-to body Ca^2+ ^current under membrane hyperpolarisation

**Figure 14:**
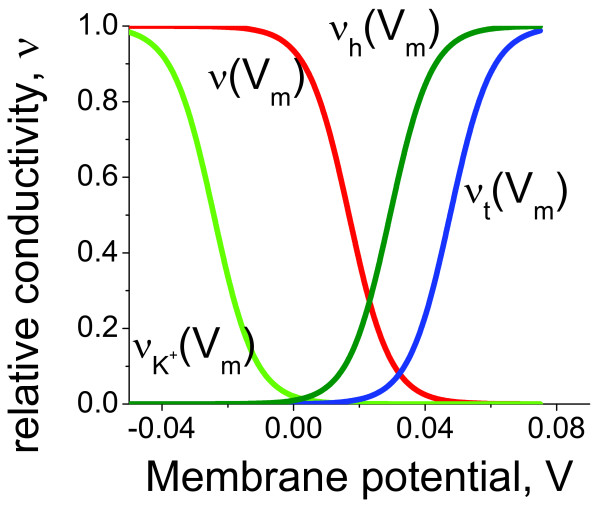
**Key ciliary ion channels conductivity dependence on membrane potential**. The overall ciliary excitability properties are due to the unique combination of ion channel conductivity dependence on membrane potential. The normalised ion channel conductivities are shown for Ca^2+ ^currents *v*(*V*), K^+ ^currents $\nu_{{\text{K}}^{+}}\left(V\right)$, hyperpolarisation-induced currents *v*_h_(*V*), and the cilia-to-cell currents *v*_t_(*V*). The hyperpolarisation and cilia-to-cell currents are activated by membrane hyperpolarisation, whereas the activation of Ca^2+ ^and K^+ ^channels takes place under depolarised conditions.

Intraciliary Ca^2+ ^concentration has been experimentally estimated to be approximately one order of magnitude higher in comparison with the intracellular levels. The Ca^2+ ^current generated by the ion flow from the ciliary compartment into the cell has been reported by a number of groups [28,52,53], but the role this current plays in the regulation of the ciliary beat remains unclear. In order to address this question, we introduced the term for this current into the equation that describes the intraciliary Ca^2+ ^concentration (equation (47) in the Methods section). In the absence of direct measurements of the dependence of conductivity on membrane potential we set the conductivity to increase in response to hyperpolarisation of membrane potential. A summary of the model responses for all channel conductivities as a function of membrane potential is shown in Figure 14. By numerically solving the coupled equations for intraciliary Ca^2+ ^and membrane potential with the cilia to the cell body contribution, we found that this current does not qualitatively change the general dynamic properties of the system.

Despite the lack of a noticeable contribution to the ciliary dynamic properties, this current requires a special consideration. Experimental studies have clearly demonstrated that intraciliary Ca^2+ ^is significantly higher than intracellular Ca^2+ ^concentration. At the same time, if the conductivity of protein structures governing the Ca^2+ ^ions movement from cilia to the body is high, most of the intraciliary ions would move from cilia into the cell body in a very short time. A simple calculation suggests that if Ca^2+ ^could freely flow from cilia into the body, the intraciliary concentration would become equal to the intracellular Ca^2+ ^concentration in less than 100 μs due to the difference in the volumes of the cell body and intraciliary compartments. Experimental measurements in ciliates show that the hyperpolarisation-induced backwards movements can last longer than 100 μseconds. It is also known that the avoidance reaction that requires long term elevation of intraciliary Ca^2+ ^concentration can be observed in hyperpolarizing solutions. During all this time the intraciliary Ca^2+ ^concentration can be several orders of magnitude higher than the intraciliary concentration. In this study, we have demonstrated that the steady-state Ca^2+ ^current under the depolarized membrane potential conditions can only be reduced by the Ca^2+^-dependent inhibition of Ca^2+ ^channels. All these observations suggest that the Ca^2+ ^removal from cilia to the cell body occurs in a membrane potential dependent manner.

### The mechanism of Ca^2+ ^and cyclic nucleotide-dependent CBF regulation

In addition to intraciliary Ca^2+ ^and K^+ ^potassium levels being coupled with the membrane potential modulation, cyclic nucleotides contribute to the regulation of one of the major ciliary beat parameters, frequency. Intraciliary Ca^2+ ^levels activate a variety of adenylate cyclases (AC) and phosphodiesterases (PDE) that produce and hydrolyse cyclic nucleotides, respectively, and thereby modulate the intraciliary cAMP and cGMP levels. At the same time, cAMP and cGMP-dependent kinases phosphorylate dynein arms [45] in the bases of cilia and thereby induce the ciliary movement [79].

**Figure 15:**
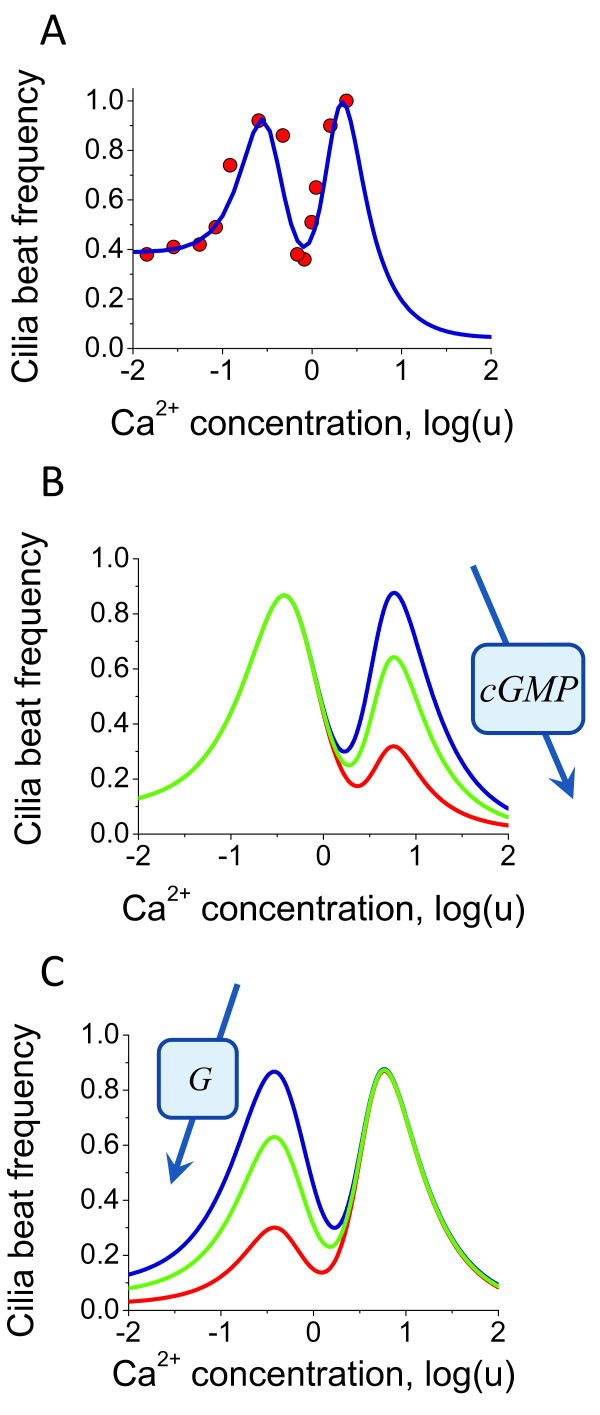
**Predictions for ciliary beat frequency modulation**. (A) The comparison of experimental data and model predictions for the CBF dependence on Ca^2+ ^concentrations. The experimental data for CBF [80] is shown as circles whereas the model prediction is represented as continuous blue line [65]. (B) GC activity depends on a number of intracellular mediators. The model describes the correlation of the right "peak" with the GC activity levels. (C) The model predicts that extracellular signals via G-protein pathway can significantly modulate the left "peak" of the plot of CBF dependence on Ca^2+ ^concentration. In both cases (B and C) the diminishing amplitude is shown as green and red lines. The model describes the distinct contribution of both internal and external signals to CBF modulation.

In a previous work we showed that the ciliary beat frequency can have a "double" bell shape dependence on Ca^2+ ^concentration [65] due to the differential regulation of adenylate and guanylate cyclase isoforms in a Ca^2+^-calmodulin (CaM) dependent manner [63,64]. One bell-shape was due to the cAMP production by a combination of AC and PDE, whereas another one was mediated by cGMP production and degradation. Our results proposed an explanation for seemingly conflicting experimental evidence suggesting that CBF can both decrease and increase with increasing Ca^2+ ^concentration. Here we extend our previous analysis and describe the conditions when one or the other peak can be significantly reduced or even disappear. Figure 15A shows the model predictions for the ciliary beat frequency in comparison with the experimental data [65,80]. Recent studies demonstrated that hormones and pharmacological agents can regulate both function and structure of cilia by interfering with the cyclic nucleotide signalling pathways [81,82]. These findings support the possibility for the development of novel therapeutic strategies for ciliary pathologies by modulating the dynamic mode of beating via ciliary membrane receptors [83].

Figure 15B and 15C demonstrate that the "amplitude" of each peak can be significantly diminished if the activity of the AC or GC, respectively is modulated by a temporary or permanent, internal or external signal. Under such a scenario, CBF can only increase or decrease if it happens to be on one slope of the bell-shaped dependence. Therefore, according to our analysis, different organisms with the same underlying ciliary regulatory system can achieve all possible CBF regulatory modes as a function of Ca^2+ ^concentration: the reverse bell-shaped dependence, if the "peak" values shown on Figure 15A occur at the lower and higher limits of the physiological range for Ca^2+ ^concentration, the bell shape dependence that can be either cAMP and cGMP dependent, and either monotonic increase or decrease if the physiological range of Ca^2+ ^concentrations occur at one of the slopes. Our model, therefore, describes the core Ca^2+^-dependent regulatory mechanisms of cilia beat, but also provides an explanation for the differences observed between cilia in different single cell organisms as well as tissue specific differences. It also unravels the mechanism for how various stimuli modulate the rate of CBF by signalling via Ca^2+^- and G-protein mediated pathways.

## Discussion

We develop a new computational model for Ca^2+ ^and membrane potential-dependent ciliary regulation that explains how different ciliary beating regimes are regulated. The model describes a novel mechanism of excitability based on the membrane potential-dependence of Ca^2+ ^currents (Figure 2) and simultaneous intraciliary Ca^2+^-concentration mediated inhibition of Ca^2+ ^channels (Figure 4). Our analysis shows that motile cilia constitute an excitable system with a novel mechanism of excitability. The ciliary system is able to generate a Ca^2+ ^spike in response to a wide range of transmembrane depolarisation (Figure 3, 5 and 9). The major difference in the ciliary excitation described here, with respect to classical excitation mechanisms, is that ciliary excitability is robust to a wide range of ionic variations in the environment.

The excitability mechanism of cells in evolutionary advanced organisms is based on a combination of the N-shaped dependence of the quick inward cationic current on the transmembrane potential and slow alterations of the K^+ ^conductivity [84-87]. The ciliary voltage-current characteristic (Figure 10H) suggests several functional dynamic modes of operation: i) single impulse generation, ii) oscillator, iii) trigger (Figure 11), all initiated by membrane depolarisation. At the same time, the hyperpolarisation-induced Ca^2+ ^currents switch the system into the mode of a monostable multivibrator, when cilia can generate a Ca^2+ ^spike in response to any alteration of membrane potential. The dynamics of such a system depends on the transmembrane potential. In other words, any alterations in the transmembrane potential (for example, initiated by variations of the external ion concentrations) switch functional performance of the system or make it non-excitable.

It was originally believed that Ca^2+^, cAMP and cGMP each represent an independent pathway of ciliary regulation, however, there is by now a significant amount of evidence that strongly suggests that all three pathways are intimately interconnected [88]. It is well established that cAMP and cGMP are synthesized by AC isoforms and hydrolysed by PDEs in a Ca^2+^-CaM-dependent manner. In this work we describe the mechanism of the cross talk between the three circuits and explain how CBF can be modulated via extra- and intraciliary pathways (Figure 15).

## Conclusions

### Therapeutic applications of systems model for intraciliary Ca^2+ ^regulation

**Figure 16:**
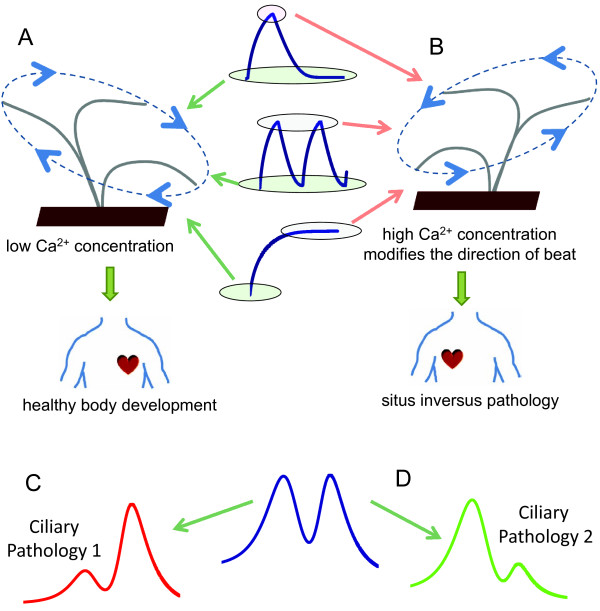
**Systems model for ciliary excitation offers new avenues for mechanistic interpretation of ciliary pathologies**. The systems model for intraciliary Ca^2+ ^regulation offers new strategies for interpretation of experimental data and development of pharmaceutical interventions for ciliary motility-associated pathologies. The ciliary system is predicted to maintain either low (A) or high (B) levels of intraciliary Ca^2+^. The extracellular conditions can shift the functional modes of ciliary activity and cause a temporal, repetitive or a long term Ca^2+ ^increase which causes cilia to reverse the direction of beat. The long term reversal of the direction of beat can explain the mechanism of the situs inversus disease, which is a congenital condition in which the major organs are mirrored with respect to their normal positions. Intraciliary Ca^2+ ^levels modulate ciliary beat frequency [65] via either an external signal through the G-protein mediated pathways or by parametric regulation of GC activity. Such alterations can represent a physiological response to external and intracellular signals, but can also occur as a result of genetic mutations. According to our model, the latter case represents a potential pathology and in either case of permanent Ca^2+^-dependent CBF alteration (C or D) requires the development of therapeutic strategies to rescue the mutation-mediated alteration of the system.

Our detailed analysis of the effects of several Ca^2+ ^and potassium currents and membrane potential on intraciliary Ca^2+ ^levels offers a new way of interpreting ciliary motility associated pathologies. There are two main Ca^2+^-mediated parameters that govern the motile function of cilia: the direction and the frequency of beat. Our model shows that there can be several dynamic regimes of intraciliary Ca^2+ ^alterations during which the intraciliary Ca^2+ ^concentration can be either at low or high levels, temporarily or for a significant period of time (Figure 16A). Experimental evidence suggests that high Ca^2+ ^reverses the direction of cilia strike (Figure 16B), and modifies the frequency in a highly nonlinear manner (Figure 15) via synthesis and hydrolysis of cyclic nucleotides (Figure 15). In a previous work [89], we showed that genetic mutations that alter the dynamic properties of a system in a permanent manner can lead to disease. In this study, we propose a conceptually similar mechanism for the pathologies associated with ciliary motility that can take place in some human diseases [6]. Our study suggests that while CBF regulation in a multicellular organism can be modulated by a number of intracellular and extracellular factors (Figure 15), genetic mutations that directly or indirectly, affect the Ca^2+^-mediated CBF dependence can dramatically impair the essential processes such as clearing function in airways, male fertility, or the determination of the left-right axis during development (Figure 16C and 16D). The treatment of the pathologies associated with this mechanism would rely on the restoration of the original Ca^2+^-dependent CBF dependence.

### Future perspective

At present there is limited understanding of the underlying biological mechanisms that govern ciliary motility. This study describes the modes of intraciliary Ca^2+ ^dynamics in a highly detailed fashion. It shows the conditions that switch the system between the modes of Ca^2+ ^spike generation, oscillatory dynamics and a trigger. The interdependent influences of Ca^2+ ^and K^+ ^currents, transmembrane potential and cyclic nucleotides modulate the ciliary beat frequency and the direction of beat in a highly nonlinear manner. The further development of mathematical models of this system is still required to represent ciliary movements as a function of Ca^2+ ^concentration and obtain the detailed understanding of ciliary motility which will be crucial for the development of new treatments for human diseases. While the core protein regulatory machinery involved in ciliary motility is very likely to be conserved, some variations in response to increased Ca^2+ ^between single cell ciliates and mammalian cilia have been reported [90]. We would argue that those differences are not due to the change in the mechanisms of Ca^2+^-dependent regulation but are rather caused by variations in the parameters of the regulatory circuits. The further investigation of single cell ciliates may allow a greater degree of characterisation of ciliary movement mechanisms, because in these systems alterations of ciliary motility translate into movement trajectories which can be easily observed.

## Methods

### Model Description

Figure 1 provides a schematic outline of the network regulating intraciliary Ca^2+ ^concentration that is considered in our model. Intraciliary Ca^2+ ^concentration is regulated by the currents of passive and active Ca^2+ ^transport, as well as by Ca^2+ ^leak into the extracellular space and into the cell body.

A basic mathematical model for intraciliary Ca^2+ ^concentration and its relationship to transmembrane potential was proposed for the first time in [91]. A large number of recent experimental findings now allow the formulation of a more advanced model that includes the crucial aspects of the molecular mechanisms governing cilia movement. Below we describe the complete model for intraciliary Ca^2+ ^regulation developed in this study.

The dynamics of intraciliary Ca^2+ ^alteration are given by:

(1) $$V_{R}\cdot \frac{d\left[Ca^{2+}\right]}{dt}=\frac{S_{R}}{z\cdot F}\cdot \left(I_{Ca^{2+}}^{P}+I_{Ca^{2+}}^{A}+I_{Ca^{2+}}^{u}\right)+I_{Ca^{2+}}^{T}+J\left(\left[Ca^{2+}\right],\left[CaM_{0}\right]\right),$$

where *V_R _- *is the cilium volume, *S*_R _- is the cilium surface area, and $I_{Ca^{2+}}^{P}$ and $I_{Ca^{2+}}^{A}$-are the Ca^2+ ^currents through the channels of passive and active Ca^2+ ^transport, respectively. $I_{Ca^{2+}}^{T}$ is the current from the cilium into the cell body. $I_{Ca^{2+}}^{u}$-is the Ca^2+ ^leakage current. $J\left(\left[Ca^{2+}\right],\left[CaM_{0}\right]\right)$ is the function that encounters Ca^2+ ^binding to and release from CaM, the main Ca^2+ ^binding protein in cilia, *z *= 2 is the Ca^2+ ^ions charge, and *F *is the Faraday constant.

The dynamics of Ca^2+ ^concentration alterations within the cilium are defined by the individual contribution of the Ca^2+ ^currents. The current via the channels of passive Ca^2+ ^transport is given by:

(2) $$I_{Ca^{2+}}^{p}=i_{Ca^{2+}}^{p}\cdot N_{Ca^{2+}}^{p},$$

where $N_{Ca^{2+}}^{p}$ is the density of functionally active Ca^2+ ^channels. $i_{Ca^{2+}}^{p}$ is the time averaged current via a single Ca^2+ ^channel. Ca^2+ ^channel activity is modulated externally by a number of metabolic pathways. Therefore, we only consider the pool of functionally active Ca^2+ ^channels. The time-averaged current via a single Ca^2+ ^channel is given by:

(3) $$i_{Ca^{2+}}^{p}=\underset{i}{\sum }g_{Ca^{2+}}^{i}\left(V_{m},Ca^{2+}\right)\cdot \left(V_{m}-E_{Ca^{2+}}\right),$$

where $g_{Ca^{2+}}^{i}\left(V_{m},Ca^{2+}\right)$ is the conductivity of a single channel in the state *i *(in the most general state Ca^2+ ^channels can have a number of states with different degrees of conductivity), $E_{Ca^{2+}}=\left(\frac{R\cdot T}{2\cdot F}\right)\cdot \ln\left(\frac{Ca_{out}^{2+}}{Ca_{in}^{2+}}\right)$ is the Ca^2+ ^potential in the equilibrium, *V_m _*is the transmembrane potential of the cilia membrane.

The Ca^2+ ^leakage current is given by:

(4) $$I_{Ca^{2+}}^{u}=g_{Ca^{2+}}^{u}\cdot \left(V_{m}-E_{Ca^{2+}}\right)$$

Assuming that the active Ca^2+ ^transport system extrudes one Ca^2+ ^ion per cycle, the current generated by the plasma membrane Ca^2+ ^pump is given by:

(5) $$I_{Ca^{2+}}^{A}=i_{A}\cdot N_{Ca^{2+}}^{A},$$

where $N_{Ca^{2+}}^{A}$ is the density of the plasma membrane Ca^2+ ^pump protein complexes bound to one Ca^2+ ^ion, and *i_A _*is the time averaged Ca^2+ ^current via a single Ca^2+ ^channel. By introducing further assumptions that all the channels of active Ca^2+ ^transport are saturated by ATP and that all bound Ca^2+ ^molecules are released into the extracellular space, this current would be defined by the dynamics of the active transport channels bound to a Ca^2+ ^ion:

(6) $$\frac{dN_{Ca^{2+}}^{A}}{dt}=k_{A}^{p}\cdot \left[Ca_{r}^{2+}\right]\cdot \left(N_{Ca^{2+}}^{00}-N_{Ca^{2+}}^{A}\right)-\left(k_{A}^{m}+k_{A}^{p}\right)\cdot N_{Ca^{2+}}^{A},$$

where $N_{Ca^{2+}}^{00}$ is the density of the active Ca^2+ ^transport channels, $k_{A}^{p}$ and $k_{A}^{m}$ are the association and dissociation constants for the Ca^2+ ^ion interaction with the active Ca^2+ ^transport channels, respectively. $k_{A}^{p}$ is the constant that defines the Ca^2+ ^ion transition from the bound state into the Ca^2+ ^channel. By introducing new non-dimensional variables: $\omega =\frac{N_{Ca^{2+}}^{A}}{N_{Ca^{2+}}^{00}},\eta =n^{m}\cdot t,k_{a}=\frac{k_{A}^{p}\cdot K_{CaM}}{n^{m}},k_{b}=\frac{k_{A}^{m}+k_{A}^{p}}{n^{m}},u=\frac{Ca^{2+}}{K_{CaM}}$, (where *n^m ^*is the dissociation constant of the protein regulating the passive Ca^2+ ^transport channels), equation (6) takes the following form:

(7) $$\frac{\mathrm{d\omega}}{\mathrm{d\eta}}=k_{a}\cdot u\cdot \left(1-\omega \right)-k_{b}\cdot \omega ,$$

The steady state-current through these active transport channels is given by:

(8) $$I_{Ca^{2+}}^{A}=\tau \cdot \frac{u}{k_{A}+u},$$

where $k_{A}=\frac{k_{A}^{m}+k_{A}^{p}}{k_{A}^{p}\cdot K_{CaM}},\tau =i_{A}\cdot N_{Ca^{2+}}^{00},u=\frac{Ca^{2+}}{K_{CaM}}$.

The model in [91] represented the leakage current from cilium into the cell body by the following expression:

(9) $$i=\vartheta \cdot \left(Ca_{r}^{2+}-Ca_{t}^{2+}\right).$$

where ϑ is the effective diffusion constant, and $Ca_{r}^{2+}$and $Ca_{t}^{2+}$ are the intraciliary and intracellular Ca^2+ ^concentrations, respectively. The Nernst equations allow a more accurate modelling of the leakage current from cilium into the cell body as follows:

(10) $$I_{Ca^{2+}}^{T}=g_{t}\left(V_{m}\right)\cdot \left(V_{rt}-\frac{R\cdot T}{2\cdot F}\cdot \ln\frac{\left[Ca_{r}^{2+}\right]}{\left[Ca_{t}^{2+}\right]}\right),$$

where *g_t_*(*V_m_*) is the overall conductivity of the cilium base area, *V_rt _*is the difference of the potential between cell body and cilia, $\left[Ca_{r}^{2+}\right]$is the intraciliary Ca^2+ ^concentration, and $\left[Ca_{t}^{2+}\right]$ is the intracellular Ca^2+ ^concentration. Equation (10) can then be represented in the following non dimensional form:

(11) $$I_{Ca^{2+}}^{T}=\beta_{1}\cdot \left(\psi_{rt}-0.5\cdot \ln\frac{u}{u_{t}}\right),$$

where $\beta_{1}=\frac{g_{t}\left(V_{m}\right)\cdot R\cdot T}{F},\psi_{rt}=\frac{V_{rt}\cdot F}{R\cdot T},u=\frac{Ca_{r}^{2+}}{K_{CaM}},u_{t}=\frac{Ca_{t}^{2+}}{K_{CaM}}$.

In the following sections we derive the models and analyse the individual contributions of the different types of Ca^2+ ^currents to the intraciliary Ca^2+ ^homeostasis.

### Model for intraciliary Ca^2+^-dependent Ca^2+ ^channel conductivity inhibition

In this model we assume that Ca^2+ ^channels located within cilia have a Ca^2+ ^binding site on the intracellular site of the channel. According to such a model, Ca^2+ ^ion binding to that site mediates the channel's transition into the closed state with no conductivity. We further assume that the characteristic time for the transition from the conductive to the non conductive states is much smaller that the characteristic Ca^2+ ^alteration times. In that case, the dynamics of the Ca^2+ ^channels transition into the closed state in response to Ca^2+ ^increase is given by:

(12) $$\frac{d\left[N\right]}{dt}=-n^{p}\cdot \left[N\right]\cdot \left[Ca^{2+}\right]+n^{m}\cdot \left(\left[N_{0}\right]-\left[N\right]\right),$$

where *N *is the number of channels in the open state, *N*_0 _is the total number of channels, *n^p ^*and *n^m ^*are the association and dissociation constants for the interaction of *Ca*^2+ ^ions with the *Ca*^2+ ^channels, respectively. The steady-state solution of equation (12) is given by:

(13) $$\left[N\right]=\left[N_{0}\right]\cdot \frac{K_{C}}{K_{C}+\left[Ca^{2+}\right]},$$

where $K_{C}=\frac{n^{m}}{n^{p}}$. In the non dimensional form this solution is given by:

(14) $$n=\frac{k_{C}}{k_{C}+u},$$

where $n=\frac{\left[N\right]}{\left[N_{0}\right]},k_{C}=\frac{K_{C}}{K_{CaM}},u=\frac{Ca^{2+}}{K_{CaM}}$.

When *u *= 0, *n *= 1 and for *u *= ∞ *n *= 0, the current via these channels equals:

(15) $$I_{Ca^{2+}}=\left[N\right]\cdot g\left(V_{m}\right)\cdot \left(V_{m}-E_{Ca^{2+}}\right).$$

The alteration of the overall conductivity of the Ca^2+ ^channels in the *Paramecium *cilia in response to the shift of transmembrane potential from *V*_0 _to *V*_1 _under the voltage clamped condition is given by:

(16) $$g\left(V,t\right)=g\left(V_{1}\right)-\left(g\left(V_{1}\right)-g\left(V_{0}\right)\right)\cdot \exp\left(\frac{t}{\tau_{p}}\right),$$

where τ*_p _*is the characteristic time of the transmembrane potential alteration from *V*_0 _to *V*_1_.

The equation for the conductivity alterations (16) can be represented as follows:

(17) g(Vm,t)=g0⋅(ν(V)1−(ν(V)1−ν(V)0)⋅exp(−tτP),

where $\psi =\frac{V\cdot F}{R\cdot T}$, *v*(*ψ, t*) is the Ca^2+ ^channel conductivity dependence on the transmembrane potential and on time.

An early study investigated the Ca^2+ ^channels' conductivity dependence on transmembrane potential *ψ *in *Paramecia *[66] and approximated it by the following equation:

(18) $$\nu \left(\psi \right)=\frac{\exp\left(\alpha \cdot \left(\psi +d\right)\right)}{\lambda +\exp\left(\alpha \cdot \left(\psi +d\right)\right)},$$

where α, *d *and λ are the parameter values that allow the best representation of the available experimental data. In this model, the steepness of the dependence of the conductivity on membrane potential is represented by the parameter α.

For simplicity we assume that the elementary intraciliary volume with inward Ca^2+ ^current does not contain any Ca^2+ ^binding proteins and Ca^2+ ^is extruded into the extracellular space by the active Ca^2+ ^transport only. The Ca^2+ ^binding to the Ca^2+ ^channels is assumed to occur much faster than the characteristic times of Ca^2+ ^channel inhibition. Under such assumptions, the intraciliary Ca^2+ ^dynamics in response to the transmembrane potential shift under the voltage clamp is given by:

(19) $$V_{R}\cdot \frac{d\left[Ca^{2+}\right]}{dt}=\frac{S_{R}}{z\cdot F}\cdot \left(-\left[N\right]\cdot \left(g\left(V_{m}\right)\cdot \left(V_{m}-E_{Ca^{2+}}\right)\right)-\beta \cdot \frac{\left[Ca^{2+}\right]}{K_{A}+\left[Ca^{2+}\right]}\right).$$

In non dimensional form, equation (19) takes the following form:

(20) $$\left\{\begin{array}{c} \frac{du}{d\eta }=s\cdot \left(\begin{array}{c} -b\cdot n\left(\eta \right)\cdot \left(\nu \left(\psi_{1}\right)-\left(\nu \left(\psi_{1}\right)-\nu \left(\psi_{0}\right)\right)\cdot \exp\left(-\frac{\eta }{\tau_{0}}\right)\right)\cdot \\ \cdot \left(\psi_{1}-0.5\cdot \ln\left(\frac{u_{out}}{u}\right)\right)-\frac{u}{k_{A}+u} \end{array}\right),\\ \frac{dn}{d\eta }=-k\cdot n\cdot u+\left(1-n\right), \end{array}\right)$$

where $u=\frac{Ca^{2+}}{K_{CaM}},u_{out}=\frac{Ca_{out}^{2+}}{K_{CaM}},k_{A}=\frac{K_{A}}{K_{CaM}},\psi =\frac{V\cdot F}{R\cdot T},\nu \left(\psi \right)=\frac{g\left(\psi \right)}{g_{0}},g_{0}=\max\left(g\left(\psi \right)\right),n\left(\eta \right)=\frac{\left[N\left(\eta \right)\right]}{\left[N_{0}\right]},\eta =n^{m}\cdot t,\tau_{0}=\tau \cdot n^{m},s=\frac{S_{R}\cdot \beta }{z\cdot F\cdot V_{R}\cdot K_{CaM}\cdot n^{m}},b=\frac{\left[N_{0}\right]\cdot g_{0}\cdot R\cdot T}{\beta \cdot F},k=\frac{K_{CaM}}{K_{C}}$

The initial conditions for the equations (20) when *η *= 0 and *ψ *= *ψ*_0 _are equal to *u*_0 _(*ψ*_0_) and *n*(*u*_0_), which can be obtained by numerical solution of these equations. The non dimensional Ca^2+ ^current is equal to:

(21) $$\begin{array}{c} i_{Ca^{2+}}=-b\cdot n\left(\eta \right)\cdot \left(\nu \left(\psi_{1}\right)-\left(\nu \left(\psi_{1}\right)-\nu \left(\psi_{0}\right)\right)\cdot \exp\left(-\frac{\eta }{\tau_{0}}\right)\right)\cdot \\ \cdot \left(\psi_{1}-0.5\cdot \ln\left(\frac{u_{out}}{u}\right)\right)-\frac{u}{k_{A}+u} \end{array}$$

### Indirect Ca^2+ ^channel conductivity regulation

The second model of intraciliary calcium regulation assumes the Ca^2+ ^binding protein interacts with the Ca^2+ ^channel and thereby causes the Ca^2+ ^channels to close. By considering that the Ca^2+ ^ion-Ca^2+ ^binding protein occurs on a faster time scale than the Ca^2+ ^binding protein-Ca^2+ ^channels interaction, the steady-state solution for the Ca^2+ ^binding protein in complex with a Ca^2+ ^ion is given by:

(22) $$\left[CaC\right]=\left[CaC_{0}\right]\cdot \frac{\left[Ca^{2+}\right]}{K_{C}+\left[Ca^{2+}\right]},$$

where [*CaC*_0_] is the total concentration of the Ca^2+ ^binding protein and $K_{C}=\;\frac{k^{m}}{k^{p}}$ is the equilibrium dissociation constant.

The Ca^2+ ^binding protein interaction with the Ca^2+ ^channels is given by:

(23) $$\frac{d\left[C\right]}{dt}=-n^{p}\cdot \left[C\right]\cdot \left[CaC\right]+n^{m}\cdot \left(\left[C_{0}\right]-\left[C\right]\right),$$

where *C *is the number of channels in the open conductive state, and *C*_0 _is the total number of channels. The steady-state solution of equation (23) is given by:

(24) $$\left[C\right]=\left[C_{0}\right]\cdot \frac{K_{CC}}{K_{CC}+\left[CaC\right]},$$

where $K_{\text{CC}}\;=\frac{{\text{n}}^{m}}{{\text{n}}^{p}}\;$ is the equilibrium dissociation constant for the Ca^2+ ^binding protein-Ca^2+ ^channels interaction. By combing equations (22) and (24) one obtains the dependence of the open Ca^2+ ^channels on the Ca^2+ ^concentration:

(25) $$c=\frac{k_{C}+u}{k_{C}+\left(cac_{0}+1\right)\cdot u},$$

where $c=\frac{\left[C\right]}{\left[C_{0}\right]},u=\frac{Ca^{2+}}{K_{CaM}},cac_{0}=\frac{CaC_{0}}{K_{CC}},k_{C}=\frac{K_{C}}{K_{CaM}}$,

Equation (23) can then be represented in the following non dimensional form:

(26) $$\frac{dc}{d\eta }=-c\cdot cac_{0}\cdot \frac{u}{k_{C}+u}+\left(1-c\right),$$

where $\eta =n^{m}\cdot t,c=\frac{\left[C\right]}{\left[C_{0}\right]},k_{C}=\frac{K_{C}}{K_{CaM}},u=\frac{Ca_{2+}}{K_{CaM}},cac_{0}=\frac{\left[CaC_{0}\right]}{K_{CC}}$,.

The solution of equation (26) that describes the dynamics of Ca^2+ ^channels in the open conductive state in response to a Ca^2+ ^shift from *u*_0 _to *u*_1 _Ca^2+ ^level is given by:

(27) $$c\left(t\right)=c^{\infty }-\left(c^{\infty }-c^{0}\right)\cdot \exp\left(-\left(cac_{0}\cdot \frac{u_{1}}{k_{C}+u_{1}}+1\right)\cdot n^{m}\cdot t\right),$$

where $c^{\infty }=\frac{k_{C}+u_{1}}{k_{C}+\left(cac_{0}+1\right)\cdot u_{1}},c^{0}=\frac{k_{C}+u_{0}}{k_{C}+\left(cac_{0}+1\right)\cdot u_{0}}$,.

The solution (27) suggests that the number of open channels would change exponentially in response to a Ca^2+ ^surge. The characteristic time for such an exponential change is given by:

(28) $$\tau_{Ca^{2+}}=\frac{k_{C}+u}{\left(k_{C}+\left(cac_{0}+1\right)\cdot u)\right)\cdot n^{m}}.$$

For cases when *cac*_0 _> > 1 and *u *> > 1, the characteristic time approximately equals $\tau_{Ca^{2+}}\approx \frac{1}{\left[CaC_{0}\right]\cdot n^{p}}.$

Under the assumption that the alteration of the transmembrane potential difference influences the Ca^2+ ^channel conductivity, the channel conductivity as a function of Ca^2+ ^concentration can be represented as follows:

(29) $$g_{Ca^{2+}}\left(t\right)=\left[C_{0}\right]\cdot g_{0}\cdot \nu \left(\psi \right)\cdot c\left(t\right).$$

When membrane potential changes from *ψ*_0 _to *ψ*_1_, and the alteration of the intraciliary Ca^2+ ^concentration is delayed, the conductivity changes from one value to another exponentially with the characteristic time *τ_V_*:

(30) $$g\left(\psi ,t\right)=g_{0}\cdot \left(\nu \left(\psi_{1}\right)-\left(\nu \left(\psi_{1}\right)-\nu \left(\psi_{0}\right)\right)\cdot \exp\left(-t∕\tau_{V}\right)\right).$$

Here we consider a simplified scenario when only passive and active Ca^2+ ^transport channels are present. For such a model, the alteration of Ca^2+ ^concentration in a cilium is given by:

(31) $$V_{R}\cdot \frac{d\left[Ca^{2+}\right]}{dt}=\frac{S_{R}}{z\cdot F}\cdot \left(I_{Ca^{2+}}^{P}+I_{Ca^{2+}}^{A}\right).$$

By substituting formulas for the passive and active Ca^2+ ^currents into equation (31), one obtains:

(32) $$V_{R}\cdot \frac{d\left[Ca^{2+}\right]}{dt}=\frac{S_{R}}{z\cdot F}\cdot \left(-\left[C_{0}\right]\cdot c\left(t\right)\cdot g_{0}\cdot \nu \left(V_{m},t\right)\cdot \left(V_{m}-E_{Ca^{2+}}\right)-\beta \cdot \frac{\left[Ca^{2+}\right]}{K_{A}+\left[Ca^{2+}\right]}\right).$$

In this equation, we include the kinetics for the active Ca^2+ ^channels due to the assumption that the dynamics of currents via the active Ca^2+ ^channels is much faster than the dynamics of currents through the passive Ca^2+ ^transport.

The non dimensional representation of Equations (32) and (23) is given by:

(33) $$\left\{\begin{array}{c} \frac{du}{d\eta }=a\cdot \left(\begin{array}{c} -b\cdot c\left(\eta ,u\right)\cdot \left(\nu \left(\psi_{1}\right)-\left(\nu \left(\psi_{1}\right)-\nu \left(\psi_{0}\right)\right)\cdot \exp\left(-\frac{\eta }{\tau_{0}}\right)\right)\cdot \\ \cdot \left(\psi_{1}-0.5\cdot \ln\left(\frac{u_{out}}{u}\right)\right)-\frac{u}{k_{A}+u} \end{array}\right),\\ \frac{dc}{d\eta }=-c\cdot cac_{0}\cdot \frac{u}{k_{C}+u}+\left(1-c\right), \end{array}\right)$$

where $u=\frac{Ca^{2+}}{K_{CaM}},u_{out}=\frac{Ca_{out}^{2+}}{K_{CaM}},k_{C}=\frac{K_{C}}{K_{CaM}},k_{A}=\frac{K_{A}}{K_{CaM}},\psi =\frac{V_{m}\cdot F}{R\cdot T},a=\frac{S_{R}\cdot \beta }{z\cdot F\cdot V_{R}\cdot K_{CaM}\cdot n^{m}},\nu \left(\psi \right)=\frac{g\left(\psi \right)}{g_{0}},g_{0}=\max\left(g\left(\psi \right)\right),c\left(\eta \right)=\frac{C\left(\eta \right)}{\left[C_{0}\right]},\eta =n^{m}\cdot t,\tau_{0}=\tau \cdot n^{m},b=\frac{\left[C_{0}\right]\cdot g_{0}\cdot R\cdot T}{\beta \cdot F},k=\frac{K_{CaM}}{K_{C}}$

By substituting the non dimensional representation of the Ca^2+ ^current (21) into (33) the equation for the intraciliary Ca^2+ ^dynamics (33) take the following form:

(34) $$\left\{\begin{array}{c} \frac{du}{d\eta }=a\cdot \left(\begin{array}{c} -b\cdot c\left(\eta ,u\right)\cdot \left(\nu \left(\psi_{1}\right)-\left(\nu \left(\psi_{1}\right)-\nu \left(\psi_{0}\right)\right)\cdot \exp\left(-\frac{\eta }{\tau_{0}}\right)\right)\cdot \\ \cdot \left(\psi_{1}-0.5\cdot \ln\left(\frac{u_{out}}{u}\right)\right)-\frac{u}{k_{A}+u}+o_{t}\left(\psi_{1}\right)\cdot \left(\psi_{st}-0.5\cdot \ln\left(\frac{u}{u_{t}}\right)\right), \end{array}\right)\\ \frac{dc}{d\eta }=-c\cdot cac_{0}\cdot \frac{u}{k_{C}+u}+\left(1-c\right), \end{array}\right)$$

where $u=\frac{Ca^{2+}}{K_{CaM}},u_{out}=\frac{Ca_{out}^{2+}}{K_{CaM}},k_{A}=\frac{K_{A}}{K_{CaM}},\psi =\frac{V_{m}\cdot F}{R\cdot T},\nu \left(\psi \right)=\frac{g\left(\psi \right)}{g_{0}},g_{0}=\max\left(g\left(\psi \right)\right),c\left(\eta ,u\right)=\frac{\left[C\left(\eta ,u\right)\right]}{\left[C_{0}\right]},\eta =n^{m}\cdot t,\tau_{0}=\tau \cdot n^{m},a=\frac{S_{R}\cdot \beta }{z\cdot F\cdot V_{R}\cdot K_{CaM}\cdot n^{m}},b=\frac{\left[C_{0}\right]\cdot g_{0}\cdot R\cdot T}{\beta \cdot F},k=\frac{K_{CaM}}{K_{C}},\nu_{t}=\frac{\beta 1}{\beta }$.

### Potassium current

Here we consider the mechanism of K^+ ^current activation by membrane potential. The full K^+ ^current in a cilium is given by:

(35) $$I_{K^{+}}=N_{K^{+}}\cdot g_{K^{+}}^{0}\cdot \nu_{K^{+}}\left(t,V_{m}\right)\cdot \left(V_{m}-E_{K^{+}}\right),$$

where $N_{K^{+}}$ is the number of open K^+ ^channels, $g_{K^{+}}^{0}$ is the maximal conductivity, and $E_{K^{+}}$ is the equilibrium K^+ ^potential.

We assume that the K^+ ^channel conductance is dependent on the membrane potential (the opposite of the case of Ca^2+ ^channel conductance). The K^+ ^channel conductance dependence on the membrane potential is given by:

(36) $$\nu_{K^{+}}\left(\psi \right)=\frac{\exp\left(\alpha_{K^{+}}\cdot \left(\psi +d_{K^{+}}\right)\right)}{\lambda_{K^{+}}+\exp\left(\alpha_{K^{+}}\cdot \left(\psi +d_{K^{+}}\right)\right)}.$$

We further assume that in response to the fast membrane potential alteration from *ψ*_0 _to *ψ*_1_, the K^+ ^conductivity is changing exponentially with a characteristic time $\tau_{K^{+}}$. In that case the conductivity dynamics over time are given by:

(37) $$\nu_{K^{+}}\left(t\right)=\nu_{K^{+}}\left(\psi_{1}\right)-\left(\nu_{K^{+}}\left(\psi_{1}\right)-\nu_{K^{+}}\left(\psi_{0}\right)\right)\cdot \exp\left(\frac{-t}{\tau_{K^{+}}}\right).$$

The parameters for the K^+ ^channels' conductivity dependence on the membrane potential can be estimated from experimental measurements of the fast K^+ ^current (10 msec) as a function of membrane potential. In such a short time K^+ ^current does not reach the maximum value and the Ca^2+ ^current is almost equal to zero. Under such conditions, the equation for the K^+ ^current is given by:

(38) $$I_{K^{+}}=i_{0}\cdot \nu_{K^{+}}\left(\psi_{m}\right)\cdot \left(\psi -\psi_{K^{+}}\right).$$

The full current measured under the voltage clamp conditions is then given by:

(39) $$\begin{array}{c} i_{P}=-b\cdot c\left(\eta ,u\right)\cdot \left(\nu \left(\psi_{1}\right)-\left(\nu \left(\psi_{1}\right)-\nu \left(\psi_{0}\right)\right)\cdot \exp\left(-\frac{\eta }{\tau_{0}}\right)\right)\cdot \left(\psi_{1}-0.5\cdot \ln\left(\frac{u_{out}}{u}\right)\right)-\\ -b_{1}\cdot \left(\nu_{K^{+}}\left(\psi_{1}\right)-\left(\nu_{K^{+}}\left(\psi_{1}\right)-\nu_{K^{+}}\left(\psi_{0}\right)\right)\cdot \exp\left(-\frac{\eta }{\tau_{K^{+}}}\right)\right)\cdot \left(\psi_{1}-\psi_{K^{+}}\right), \end{array}$$

where $b_{1}=N_{K^{+}}\cdot \frac{g_{K^{+}}^{0}}{g_{0}}$.

### The transmembrane potential dynamics

All the models described above were specifically developed under the voltage clamp conditions. However, Ca^2+ ^as well as K^+ ^currents can alter the membrane potential. In order to account for this effect, we considered the membrane potential dynamics in the absence of voltage clamp. Having established that the main contributors to the registered currents are the Ca^2+ ^and K^+ ^currents, we analyse the membrane potential dynamics as a function of Ca^2+ ^and K^+ ^current contributions:

(40) $$C_{m}\cdot \frac{dV_{m}}{dt}=I_{Ca^{2+}}+I_{K^{+}}.$$

In the non dimensional form the equation for the membrane potential dynamics is given by:

(41) $$\frac{d\psi }{d\eta }=\rho \cdot \left(\begin{array}{c} -b\cdot \left(c\left(\eta ,u\right)\cdot \nu \left(\eta ,\psi \right)+\nu_{Ca^{2+}}^{st}\right)\cdot \left(\psi -0.5\cdot \ln\left(\frac{u_{out}}{u}\right)\right)-\\ -b_{1}\cdot \left(\nu_{K^{+}}\left(\eta ,\psi \right)+\nu_{K^{+}}^{st}\right)\cdot \left(\psi -\psi_{K^{+}}\right) \end{array}\right),$$

where $\psi =\frac{V_{m}\cdot F}{R\cdot T},u=\frac{\left[Ca^{2+}\right]}{K_{CaM}},\nu_{Ca^{2+}}^{st}$, and $\nu_{K^{+}}^{st}$ are the steady-state Ca^2+ ^and K^+ ^channel conductivities, respectively, $\eta =n^{m}\cdot t,\rho =\frac{g^{0}}{C_{m}\cdot n^{m}}$,.

In this equation, we take into consideration the contribution of the independent parts of the Ca^2+ ^and K^+ ^currents, conductivities $\nu_{Ca^{2+}}^{st}$ and $\nu_{K^{+}}^{st}$, respectively, that do not depend on membrane potential or Ca^2+ ^concentration. We also use the fact that Ca^2+ ^and K^+ ^channel conductivities change on a much faster time scale in comparison with membrane potential or Ca^2+ ^concentration. Such an approximation allows us to employ the steady-state solutions for the channel conductivities as a function of membrane potential and Ca^2+ ^concentration. The described assumptions and considerations lead to the following system of two nonlinear coupled equations for intraciliary Ca^2+ ^concentration and membrane potential:

(42) $$\left\{\begin{array}{c} \frac{du}{d\eta }=s\cdot \left(-b\cdot \left(c\left(u\right)\cdot \nu \left(\psi \right)+\nu_{Ca^{2+}}^{st}\right)\cdot \left(\psi -0.5\cdot \ln\left(\frac{u_{out}}{u}\right)\right)-\frac{u}{k_{A}+u}\right),\\ \frac{d\psi }{d\eta }=-\rho \cdot \left(\begin{array}{c} -b\cdot \left(c\left(u\right)\cdot \nu \left(\psi \right)+\nu_{Ca^{2+}}^{st}\right)\cdot \left(\psi -0.5\cdot \ln\left(\frac{u_{out}}{u}\right)\right)-\\ -b_{1}\cdot \left(\nu_{K^{+}}\left(\psi \right)+\nu_{K^{+}}^{st}\right)\cdot \left(\psi -\psi_{K^{+}}\right)+I_{0} \end{array}\right), \end{array}\right)$$

where *I*_0 _is the non dimensional inward current.

### Currents activated by the membrane hyperpolarisation

The membrane hyperpolarisation current is given by:

(43) $$I_{Ca^{2+}}^{ht}=N_{Ca^{2+}}^{ht}\cdot g_{Ca^{2+}}^{ht}\left(\psi ,t\right)\cdot \left(V_{m}-E_{Ca_{t}^{2+}}\right)$$

where $N_{Ca^{2+}}^{ht}$ is the number of open, membrane hyperpolarisation-activated Ca^2+ ^channels located on the cell body, $g_{Ca^{2+}}^{ht}$is the conductivity of the channel, and $E_{Ca_{t}^{2+}}$ is the equilibrium potential for Ca^2+ ^ions.

(44) $$g_{Ca^{2+}}^{ht}\left(\psi ,t\right)=g^{0}\cdot \nu_{h}\left(\psi ,t\right),$$

where $g^{0}=\max\left(\overset{ht}{\underset{Ca^{2+}}{g}}\left(\psi ,t\right)\right),\nu_{h}\left(\psi \right)=\frac{\exp\left(\alpha_{h}\cdot \left(\psi +d_{h}\right)\right)}{\lambda_{h}+\exp\left(\alpha_{h}\cdot \left(\psi +d_{h}\right)\right)}$

By combining the contribution from different types of currents, one can derive the dynamics of the membrane potential alteration as follows:

(45) $$\begin{array}{c} C_{m}\cdot \frac{dV_{m}}{dt}=n_{r}\cdot \left(\left(-N_{Ca^{2+}}^{0}\cdot \left(w_{1}\cdot g_{Ca^{2+}}^{1}+w_{3}\cdot g_{Ca^{2+}}^{3}\right)+N_{Ca^{2+}}^{ht}\cdot g_{Ca^{2+}}^{ht}\right)\cdot \left(V_{m}-\frac{R\cdot T}{2\cdot F}\cdot \ln\left(\frac{u_{out}}{u_{t}}\right)\right)\right)-i_{A}\cdot N_{Ca^{2+}}^{00}\cdot \omega +\\ +\left(N_{K^{+}}^{01}\cdot {ḡ}_{K^{+}}^{1}\left(t,V_{m}\right)+N_{K^{+}}^{02}\cdot {ḡ}_{K^{+}}^{2}\left(t,V_{m}\right)+N_{K^{+}}^{03}\cdot {ḡ}_{K^{+}}^{11}\left(V_{m}\right)\cdot \nu_{1}+N_{K^{+}}^{04}\cdot {ḡ}_{K^{+}}^{22}\left(V_{m}\right)\cdot \nu_{2}\right)\cdot \left(V_{m}-E_{K^{+}}\right)-I_{K^{+}}^{A}. \end{array}$$

The coupled system of differential equations for the Ca^2+ ^and membrane potential dynamics can be further formulated as:

(46) $$\left\{ \begin{array}{l} \frac{du}{d\eta }=s\cdot \left(-b\cdot \left(c\left(u\right)\cdot \nu_{Ca^{2+}}\left(\psi \right)+\nu_{Ca^{2+}}^{st}\right)\cdot \left(\psi -0.5\cdot \ln\left(\frac{u_{out}}{u}\right)\right)-\frac{u}{k_{A}+u}\right),\\ \frac{d\psi }{d\eta }=-\rho \cdot \left(\left(-b\cdot (c\left(u\right)\cdot \nu \left(\psi \right)+\nu_{Ca^{2+}}^{st}\right)-\nu Cah\cdot \nu_{h}\left(\psi \right)\right)\cdot \left(\psi -0.5\cdot \ln\left(\frac{u_{out}}{u_{t}}\right)\right)\\ -b_{1}\cdot \left(\nu_{K^{+}}^{1}\left(\psi \right)+\nu_{Kca}\cdot \nu_{K^{+}}^{2}\left(\psi ,u\right)+\nu_{K^{+}}^{st}\right)\cdot \left(\psi -\psi_{K^{+}}\right)+I_{0}, \end{array}\right.-$$

where *v_h_*(*ψ*) is the Ca^2+ ^current contribution, activated by membrane depolarization, and $\nu_{K^{+}}\left(\psi ,u\right)$ is the Ca^2+^-dependent K^+ ^current contribution.

### Cilia-to body Ca^2+ ^current

The incorporation of the Ca^2+ ^current from cilia to cell into the equations for the intraciliary Ca^2+ ^and membrane potential system leads to an additional term being added to the equations (46):

(47) $$\begin{array}{c} \frac{du}{d\eta }=s\cdot \left(\begin{array}{c} -b\cdot \left(c\left(u\right)\cdot \nu \left(\psi \right)+\nu_{Ca^{2+}}^{st}\right)\cdot \left(\psi -0.5\cdot \ln\left(\frac{u_{out}}{u}\right)\right)-\\ -\frac{u}{k_{A}+u}+\nu_{Cat}\cdot \nu_{t}\left(\psi \right)\cdot \left(\psi_{tr}-0.5\cdot \ln\left(\frac{u}{u_{t}}\right)\right) \end{array}\right),\\ \frac{d\psi }{d\eta }=-\rho \cdot \left(-b\cdot \left(c\left(u\right)\cdot \nu \left(\psi \right)+\nu_{Ca^{2+}}^{st}\right)-\nu_{Cah}\cdot \nu_{h}\left(\psi \right)\right)\cdot \left(\psi -0.5\cdot \ln\left(\frac{u_{out}}{u_{t}}\right)\right)-\\ -b_{1}\cdot \left(\nu_{K^{+}}\left(\psi \right)+\nu_{Kca}\cdot \nu_{K^{+}}^{1}\left(u,\psi \right)+\nu_{K^{+}}^{st}\right)\cdot \left(\psi -\psi_{K^{+}}\right)+I_{0}, \end{array}$$

In the general case the conductivity of the protein complexes governing the Ca^2+ ^current from cilia to the cell body depends on the membrane potential:

(48) $$\nu_{t}\left(\psi \right)=\frac{\exp\left(\alpha_{t}\cdot \left(\psi +d_{t}\right)\right)}{\lambda_{t}+\exp\left(\alpha_{t}\cdot \left(\psi +d_{t}\right)\right)}.$$

All parameter values used in the above equations are given in Table 1. The relationship between dimensional and non-dimensional quantities for Ca^2+ ^concentration and membrane potential are given in Table 2.

**Table 1:** Parameter values employed in the systems model for the ciliary excitation

Parameter	Value (dimensionless unless otherwise stated)	Figure No	Equation
*α*	4	2A	18

*d*	0.4	2A	18

*λ*	0.5	2A	18

*b*	2	2B, 3, 5, 6, 7, 9, 10	20, 21, 34, 39

*k_A_*	1	2B, 3, 5, 6, 7, 11	20, 34, 42

*u_out_*	1000	2B, 3, 5, 6, 7	20, 21, 34

*ψ*_0_	-1.2	2B, 3, 5, 6, 7, 8	20, 21, 34, 39

*V*_0_	30 mV	2B, 3, 5, 6, 7, 8	20, 21, 34, 39

*ψ*_1_	-1, -0.8, -0.5, -0.2, 0, 0.2, 0.5	2B, 3, 5, 6, 7, 8	20, 21, 34, 39

*V*_1_	25, 20, 12.5, 5, 0, -5, -12.5 mV	2B, 3, 5, 6, 7, 8	20, 21, 34, 39

*s*	0.5	2B, 3, 5, 6, 7	20

*τ*_0_	0.02	2B, 3, 5, 6, 7, 9, 10	20, 34, 39

*k*	2	2B, 3, 5, 6, 7	20

*K_C_*	1	4, 5, 6, 7	25

*CaC*_0_	5, 10, 50, 100	4, 5, 6, 7	25, 34

*a*	4	7, 11	34, 42

$\alpha_{K^{+}}$	0.5	8, 10	36

$\lambda_{K^{+}}$	0.005	8, 10	36

$d_{K^{+}}$	0.5	8, 10	36

*b*_1_	1	9	39

*b*	8	11, 12, 13, 14	42, 46

*cac*_0_	20	11, 12, 13, 14	42

$\nu_{Ca^{2+}}^{st}$	0.01	11, 12, 13, 14	42, 46

$\nu_{K^{+}}^{st}$	0.01	11, 12, 13, 14	42, 46

*ρ*	10	11, 12, 13, 14	42, 46

*s*	0.5	11, 12, 13, 14	42, 46

*vCah*	0.9	12, 13, 14	46

*λ_h_*	5	12, 13, 14	44

*d_h_*	1	12, 13, 14	44

*α_h_*	4	12, 13, 14	44

**Table 2:** The relationship between dimensional and non-dimensional quantities for Ca^2^^+ ^concentration and membrane potential

Variables	Dimensional variables	Non-dimensional variables	Coefficient value
**Calcium**	*Ca*^2+ ^(M/L)	$u=\frac{Ca^{2+}}{K_{CaM}}$	*K_CaM _*= 4 *μM*

**Transmembrane** **potential**	*V_m_*	$\psi_{m}=\frac{F\cdot V_{m}}{R\cdot T}$	$\frac{RT}{F}=-0.025\;V$

## Authors' contributions

NVK, ANG, BG, MZQC, ID, CH and AA developed and implemented the project under the supervision of DGB, RNK, YU and NVV. XY, SKS and CMS contributed to the analysis of the model. All authors contributed to the writing of the final manuscript. All authors read and approved the final manuscript.
